# Asymmetric Entanglement-Assisted Quantum MDS Codes Constructed from Constacyclic Codes

**DOI:** 10.3390/e25121603

**Published:** 2023-11-30

**Authors:** Jianzhang Chen, Wanchuan Fang, Shuo Zhou, Jie Qiu, Chenyang Zhang, Yixin Xu, Bozhe Zeng, Youqin Chen

**Affiliations:** 1 College of Computer and Information Sciences, Fujian Agriculture and Forestry University, Fuzhou 350002, China; jzchen@fafu.edu.cn (J.C.);; 2 College of Computer Science and Mathematics, Fujian University of Technology, Fuzhou 350118, China

**Keywords:** asymmetric entanglement-assisted quantum codes, constacyclic codes, maximal-distance separable codes

## Abstract

Due to the asymmetry of quantum errors, phase-shift errors are more likely to occur than qubit-flip errors. Consequently, there is a need to develop asymmetric quantum error-correcting (QEC) codes that can safeguard quantum information transmitted through asymmetric channels. Currently, a significant body of literature has investigated the construction of asymmetric QEC codes. However, the asymmetry of most QEC codes identified in the literature is limited by the dual-containing condition within the Calderbank-Shor-Steane (CSS) framework. This limitation restricts the exploration of their full potential in terms of asymmetry. In order to enhance the asymmetry of asymmetric QEC codes, we utilize entanglement-assisted technology and exploit the algebraic structure of cyclotomic cosets of constacyclic codes to achieve this goal. In this paper, we generalize the decomposition method of the defining set for constacyclic codes and apply it to count the number of pre-shared entangled states in order to construct four new classes of asymmetric entanglement-assisted quantum maximal-distance separable (EAQMDS) codes that satisfy the asymmetric entanglement-assisted quantum Singleton bound. Compared with the codes existing in the literature, the lengths of the constructed EAQMDS codes and the number of pre-shared entangled states are more general, and the codes constructed in this paper have greater asymmetry.

## 1. Introduction

Quantum computing is a new computing paradigm that follows the laws of quantum mechanics to manage the processing of quantum information units. Compared with traditional computing, quantum computing offers exponential acceleration and significant breakthroughs [1]. In recent years, various scientifically advanced countries have placed great emphasis on quantum computing research and have launched their own quantum information technology strategies. They aim to capture the leading edge of the next wave of technological development and strive to achieve “quantum supremacy” as soon as possible [1]. Among them, Google, Rigetti Computing, and other world-leading institutions are engaged in quantum computing research. All of this indicates that quantum computing research is a very important and worthwhile field of study. It is particularly noteworthy that A. S. Holevo received the prestigious Claude E. Shannon Award from the IEEE Information Theory Society in 2016, in recognition of his groundbreaking contributions to quantum information theory. This honor marks the first time that research in the field of quantum information theory has been recognized with the highest award in the field of information theory. Additionally, quantum coding theory is an important research topic in quantum Shannon theory.

One of the key technologies in quantum computing research is QEC codes [2], which are essential for protecting quantum information from quantum decoherence caused by quantum noise during quantum computing and communication processes. Designing high-performance QEC codes is crucial for the future realization of quantum computing and communication. Classical computers can significantly enhance the reliability of computer communication and approach the Shannon capacity of channels with the help of error-correcting codes. However, due to the uncloneability of quantum states, the continuity of quantum errors, and the destruction of quantum information upon measurement [2], existing classical error-correcting code technology cannot be directly applied to protect quantum information. Research studies have shown that QEC codes can protect stored quantum information and also enable fault-tolerant quantum gate operations, fault-tolerant quantum state preparation, and fault-tolerant quantum measurements, thereby making quantum information processing reliable in noisy environments [2]. Currently, the research on QEC codes has become one of the important topics in the field of quantum computing and quantum information.

Similar to classical error-correcting codes theory, constructing and searching for QEC codes with maximum minimum distance is a core problem in the study of quantum coding theory. The quantum CSS code model, which transforms complex physical quantities into mathematical models, provides a connection between QEC codes and classical error-correcting codes [3]. Although a quantum CSS framework can be constructed from classical codes that are dual-containing (self-orthogonal), constructing quantum maximal-distance separable (MDS) codes with a relatively large minimum distance is not an easy task. Except for some special lengths of quantum codes, the minimum distance of most known *q*-ary quantum MDS codes is less than or equal to $\frac{q}{2}+1$. Currently, the methods used to construct quantum MDS codes mainly adopt cyclic codes, constacyclic codes (narrow or non-narrow [4]) over finite fields, as well as generalized Reed–Solomon (RS) codes. Compared to traditional cyclic codes over finite fields, constacyclic codes over finite fields are a generalization of cyclic codes. They not only integrate the good performance of cyclic codes but also exhibit several new characteristics and rich algebraic structures. Additionally, compared to generalized Reed–Solomon codes over finite fields, constacyclic codes have a simpler algebraic structure of cyclotomic cosets and demonstrate good error-correcting capabilities. Although constacyclic dual-containing codes have better parameters and can obtain a few quantum MDS codes with a minimum distance greater than $\frac{q}{2}+1$, the minimum distance of quantum MDS codes constructed by constacyclic codes is not always greater than $\frac{q}{2}+1$. Additionally, the minimum distance of traditional quantum MDS codes still cannot exceed the q+1 limit. Therefore, the dual-containing condition has posed an obstacle to the development of quantum coding theory [5].

In recent years, a major breakthrough in the field of QEC codes has been the construction system of entanglement-assisted stabilizer codes, which allows two parties to share a certain number of maximally entangled pairs in advance. This removes the restriction that the quantum stabilizer must be a subgroup of the Pauli group, thus allowing the error correction properties of any non-Abelian group to be applied [2]. The proposal of entanglement-assisted quantum error-correcting (EAQEC) codes breaks the limitation of the dual-containing condition previously required by stabilizer codes, which alleviates the challenges in determining the dual-containing condition necessary for modern codes such as LDPC codes [6] and Turbo codes [7]. Due to the more extensive algebraic structure of constacyclic codes over finite fields compared to cyclic codes, the synergy of entanglement-assisted techniques and constacyclic codes provides a rich resource for exploring the construction of entanglement-assisted quantum MDS (EAQMDS) codes. However, entanglement-assisted techniques also require the consumption of additional entanglement resources. Therefore, for EAQMDS codes, it is of significant research importance and potential application value to construct more quantum MDS codes with a minimum distance greater than $\frac{q}{2}+1$ or even q+1, while consuming entanglement resources as little as possible.

QEC codes defined over quantum channels—where qubit-flip errors (qudit-flip errors are primarily considered for high-dimensional systems) and phase-shift errors may have different probabilities—are called asymmetric QEC codes [8]. In many quantum mechanical systems, the probabilities of occurrence of qubit-flip and phase-shift errors are quite different [2]. Previous research on constructing entanglement-assisted quantum codes has predominantly focused on code constructions for symmetric quantum channels [9,10,11,12,13,14,15]. Recently, Galindo et al. introduced the concept of asymmetric EAQEC codes and presented an explicit computation of the parameters for asymmetric EAQEC codes derived from BCH codes [16]. Moreover, the quantum Singleton bound for asymmetric EAQMDS codes has been established in [16,17,18,19], along with the proposal of a general Euclidean construction method based on cyclic codes. Although there has been some progress in the study of asymmetric EAQMDS codes, several issues still need to be addressed.

- Although sharing a certain number of entangled states between the communicating parties can enhance the error-correcting ability of quantum codes, it actually incurs higher entanglement preparation costs. Exploring how to obtain asymmetric EAQMDS codes with either good error-correcting capability or a high code rate, within the context of lower-cost preparation of entangled resources, is a topic worth exploring.
- Although constacyclic codes inherit the good performance of cyclic codes, a pertinent question is how we can utilize the decomposition method of the defining set of constacyclic codes to achieve a general number of entangled states for the constructed EAQMDS codes?

Therefore, asymmetric EAQMDS codes are constructed in this paper by using constacyclic codes over finite fields. The main contributions are as follows:

- The decomposition method of the defining set of constacyclic codes is re-characterized and applied to construct asymmetric EAQMDS codes. Under the same code length and fixed $d_{z}$ and $d_{x}$, some asymmetric EAQMDS codes constructed in this paper can achieve the same net rate (k−c)/n as the quantum codes in [18]. However, the asymmetric EAQMDS codes in this paper require fewer entangled states, which means that the preparation cost and cost of quantum entangled states required in this paper are less. In addition, some quantum codes that do not exist in [18,20,21,22,23,24,25,26,27,28,29] are obtained in this paper. Moreover, the minimum distance $d_{z}$ of some asymmetric EAQMDS codes constructed in this paper is much greater than *q*, indicating that such quantum codes have greater asymmetric error-correcting ability.
- The number of entangled states in the constructed quantum codes exhibits generality. The parameters of the constructed asymmetric EAQMDS codes in this paper are listed as follows:
  (1) ${\left[\left[\frac{q^{2}+1}{\beta },\frac{q^{2}+1}{\beta }-2\left(\delta_{1}+\delta_{2}+1-v\right),2\delta_{2}+2/2\delta_{1}+2;2v\right]\right]}_{q^{2}}$, where q=2βm+β+t is an odd prime power with a positive integer *m* and t≥2 is even, such that $\beta =t^{2}+1$, $0\leq \delta_{1}\leq \frac{q-\beta -t}{2\beta }$, $\frac{q+\beta -t}{2\beta }\leq \delta_{2}\leq \frac{q^{2}+1}{2\beta }-q\delta_{1}+vq-1$ and $0\leq v\leq \delta_{1}$.
  (2) ${\left[\left[\frac{q^{2}+1}{\beta },\frac{q^{2}+1}{\beta }-2\left(\delta_{1}+\delta_{2}+1-v\right),2\delta_{2}+2/2\delta_{1}+2;2v\right]\right]}_{q^{2}}$, where q=2βm+β−t is an odd prime power with a positive integer *m* and t≥2 is even, such that $\beta =t^{2}+1$, $0\leq \delta_{1}\leq \frac{q-\beta +t}{2\beta }$, $\frac{q+\beta +t}{2\beta }\leq \delta_{2}\leq \frac{q^{2}+1}{2\beta }-q\delta_{1}+vq-1$ and $0\leq v\leq \delta_{1}$.
  (3) ${\left[\left[\frac{2(q^{2}+1)}{\alpha },\frac{2(q^{2}+1)}{\alpha }-2\left(\delta_{1}+\delta_{2}+1-v\right),2\delta_{2}+2/2\delta_{1}+2;2v\right]\right]}_{q^{2}}$, where q=αm+α+t is an odd prime power with a positive integer *m* and t≥3 is odd, such that $\alpha =t^{2}+1$, $0\leq \delta_{1}\leq \frac{q-t}{\alpha }$ and $\frac{q+\alpha -t}{\alpha }\leq \delta_{2}\leq \frac{q^{2}+1}{\alpha }-q\delta_{1}+vq-1$, and $0\leq v\leq \frac{t-1}{2}$.
  (4) ${\left[\left[\frac{2(q^{2}+1)}{\alpha },\frac{2(q^{2}+1)}{\alpha }-2\left(\delta_{1}+\delta_{2}+1-v\right),2\delta_{2}+2/2\delta_{1}+2;2v\right]\right]}_{q^{2}}$, where q=αm+α−t is an odd prime power with a positive integer *m* and t≥3 is odd, such that $\alpha =t^{2}+1$, $0\leq \delta_{1}\leq \frac{q+t}{\alpha }$ and $\frac{q+\alpha +t}{\alpha }\leq \delta_{2}\leq \frac{q^{2}+1}{\alpha }-q\delta_{1}+vq-1$, and $0\leq v\leq \frac{t-1}{2}$.

The rest of this paper is organized as follows. In Section 2, we present some related works about symmetric and asymmetric EAQEC codes. In Section 3, we present some definitions and basic results of constacyclic codes and asymmetric EAQEC codes. In Section 4, four families of EAQMDS codes are constructed by using constacyclic codes with lengths of $\frac{q^{2}+1}{\beta }$ and $\frac{2(q^{2}+1)}{\alpha }$, respectively, where the number of entangled states constructed for asymmetric EAQMDS codes is universal. In Section 5, we mainly compare the parameters of the asymmetric EAQMDS codes constructed in this paper with those of the quantum codes in [18,25,27,28]. In Section 6, we present our conclusion and discussion.

## 2. Related Works

Since the works of Shor [30] and Steane [2,31], QEC codes have attracted a lot of attention from experts and scholars. Constructing good quantum codes through classical codes is particularly important in quantum information and quantum computing [32,33,34,35,36]. Some scholars utilize constacyclic codes (including both cyclic and negacyclic codes) to construct quantum MDS codes with a minimum distance greater than $\frac{q}{2}+1$ based on Hermite construction. Kai et al. used negacyclic codes to construct two classes of quantum MDS codes [37]. Since then, other types of negacyclic or constacyclic codes have also been studied by scholars, as detailed in references [5,38,39,40,41,42,43,44]. Although quantum MDS codes can be obtained through the construction of dual-containing (self-orthogonal) classical codes, most known *q*-ary quantum MDS codes have a minimum distance that is less than or equal to $\frac{q}{2}+1$. Therefore, the dual-containing condition restricts the development of quantum coding theory [5].

Recently, the discovery of EAQEC coding theory has played an important role in the field of quantum information and quantum computing. Brun et al. proposed the entanglement-assisted stabilizer framework in [45]. They showed that some EAQEC codes can be constructed without the need for the dual-containing condition of classical quaternary codes if a certain number of entangled states are pre-shared between the sender and receiver. In [46], Li et al. proposed the decomposition method of the defining set of cyclic codes and used this method to construct EAQEC codes with good parameters. In [25], Fan et al. constructed EAQMDS codes with the help of a small number of pre-shared maximally entangled states. Guenda et al. introduced the concept of the Hull of classical codes in [47] and used it to construct some EAQMDS codes. In fact, the concept of the decomposition method of the defining set is functionally equivalent to the concept of the Hull of classical codes, both aimed at counting the entangled states of entanglement-assisted quantum codes. In [48], we proposed the concept of the decomposition method of the defining set for negacyclic codes over finite fields and employed it to construct several classes of EAQMDS codes of varying lengths. In [5,49], Lü et al. constructed several classes of EAQMDS by using negacyclic and constacyclic codes with the decomposition method of the defining set, and some of the EAQMDS codes have minimum distances greater than q+1. In [50], constacyclic codes with a length of $\frac{q^{2}-1}{r}$ were used to construct some new EAQMDS codes, where r=3,5,7, and q≡1 mod 5. In fact, pre-shared entangled states can improve the error correction ability of quantum codes. Quantum MDS codes that originally have a minimum distance of less than $\frac{q}{2}+1$ can have their minimum distance increased to more than $\frac{q}{2}+1$ or even q+1 through the pre-shared entangled states. Therefore, it is necessary to consider EAQMDS codes with a larger minimum distance, and it is worth exploring how to count pre-shared entangled states to achieve a minimum distance greater than $\frac{q}{2}+1$ or even q+1 for quantum MDS codes.

In quantum channels, the probabilities of qubit-flip (or qudit-flip) and phase-shift errors can be significantly different, and QEC codes that take advantage of this asymmetry are called asymmetric QEC codes. In 2007, Ioffe et al. [8] proposed asymmetric QEC schemes based on BCH and LDPC codes. Since then, some good asymmetric QEC codes have been constructed from families of known classical codes, such as BCH codes [51,52,53], constacyclic codes [54,55,56], RS codes [57], and others. New families of non-binary asymmetric quantum BCH codes and subsystem BCH codes were constructed by Leng and Ma [58]. The bounds for asymmetric quantum stabilizer codes were provided by Sarvepalli et al. [59,60]. Tang et al. [61] constructed several classes of asymmetric QEC codes with unbounded lengths from repeated-root cyclic codes and proposed asymmetric QEC codes that exceeded the asymmetric quantum Gilbert–Varshamov bound [62]. In [63], a few of the good asymmetric QEC codes were obtained from quasi-cyclic codes over small fields, which cannot all be deduced by the asymmetric quantum Gilbert–Varshamov bound in Matsumoto [62]. In addition, there are other construction methods that have also promoted the development of the theory of asymmetric QEC codes [64,65,66]. Recently, Galindo et al. [16] introduced the concept of asymmetric EAQEC codes and provided a Gilbert–Varshamov bound for asymmetric EAQEC codes. They then presented the explicit computation of the parameters of asymmetric EAQEC codes derived from BCH codes. In [18], the authors established a bound for pure asymmetric EAQEC codes similar to the quantum Singleton bound and introduced the definition of pure asymmetric EAQMDS codes. They then constructed three new families of asymmetric EAQEC codes. In [17], the authors determined the number of maximal entangled states required for asymmetric EAQEC codes by constructing linear *l*-intersection pairs for MDS codes. Compared to cyclic codes over finite fields, constacyclic codes have significant algebraic structural advantages, but there are currently few references on the use of constacyclic codes to construct asymmetric EAQEC codes, especially asymmetric EAQMDS codes.

## 3. Preliminaries

### 3.1. Constacyclic Codes

In this subsection, we recall some basic results about constacyclic codes in [33,37,38,67,68,69].

Let $F_{q^{2}}$ be the finite field with $q^{2}$ elements, where *q* is a power of *p* and *p* is an odd prime number. $F_{q^{2}}^{n}$ is the *n*-dimension row vector space over $F_{q^{2}}$, in which *n* is a positive integer. An ${\left[n,k,d\right]}_{q^{2}}$ linear code of length *n* over finite field $F_{q^{2}}$ is a nonempty subspace of $F_{q^{2}}^{n}$ and its minimum distance is *d*. Throughout this paper, we assume that *n* is a positive integer relatively prime to *q*, i.e., gcd(n,q)=1. Moreover, the Singleton bound of linear codes is given as follows.

**Proposition 1**  ([67,70])**.** *(Singleton bound) If an [n,k,d] linear code C over $F_{q^{2}}$ exists, then*

(1) k≤n−d+1.

*If k=n−d+1, then C is called an MDS code.*

Let $a^{q}=\left(a_{0}^{q},a_{1}^{q},\ldots ,a_{n-1}^{q}\right)$ denote the conjugation of the vector $a=(a_{0},a_{1},\ldots ,a_{n-1})$. For $u=(u_{0},u_{1},\ldots ,u_{n-1})$ and $v=\left(v_{0},v_{1},\ldots ,v_{n-1}\right)\in F_{q^{2}}^{n},$ the Hermitian inner product is defined as ${\langle u,v\rangle }_{h}=u_{0}v_{0}^{q}+u_{1}v_{1}^{q}+\ldots +u_{n-1}v_{n-1}^{q}.$ The Hermitian dual code of C can be defined as $C^{{⊥}_{h}}=\left\{u\in F_{q^{2}}^{n}|{\langle u,v\rangle }_{h}=0\;for\;all\;v\in C\right\}.$ If $C\subseteq C^{{⊥}_{h}},$ then C is called Hermitian self-orthogonal code. If $C^{{⊥}_{h}}\subseteq C,$ then C is a Hermitian dual-containing code.

For a nonzero element $\lambda \in F_{q^{2}}^{*}$, a linear code C of length *n* over $F_{q^{2}}$ satisfies the property that

(2) $$\left(c_{0},c_{1},\ldots ,c_{n-1}\right)\in C\Rightarrow \left(\lambda c_{n-1},c_{0},c_{1},\ldots ,c_{n-2}\right)\in C,$$

then C is called a $q^{2}$-ary λ-constacyclic code of length *n* over $F_{q^{2}}$. When λ=−1, C is a negacyclic code. When λ=1, C is a cyclic code.

From [33,37], a $q^{2}$-ary λ-constacyclic code C over $F_{q^{2}}$ of length *n* is precisely an ideal in $F_{q^{2}}\left[x\right]/\langle x^{n}-\lambda \rangle$ and C can be generated by a monic polynomial g(x) which divides $x^{n}-\lambda$. Assume that $\lambda \in F_{q^{2}}^{*}$ is a primitive *r*-th root of unity, and then it exists a primitive rn-th root of unity over some extension field of $F_{q^{2}}$, denoted by η, such that $\eta^{n}=\lambda .$ Let $\xi =\eta^{r}$, then ξ is a primitive *n*-th root of unity, and the roots of $x^{n}-\lambda$ are precisely the elements $\eta \xi^{i}=\eta^{1+ri}$, where 0≤i≤n−1. Set $O_{rn}=\left\{1+ri|0\leq i\leq n-1\right\}$. If C is a λ-constacyclic code over $F_{q^{2}}$ of length *n* with generator polynomial g(x), then the defining set of the constacyclic code C=〈g(x)〉 is the set $Z=\{i\in O_{rn}|\eta^{i}\;is\;a\;root\;of\;g\left(x\right)\}.$ For each $i\in O_{rn}$, the set $C_{i}=\left\{i,iq^{2},iq^{4},\ldots ,iq^{2k-2}\right\}\;\mathrm{mod}\;rn$ is called the $q^{2}$-cyclotomic coset modulo rn, where *k* is the smallest positive integer such that $iq^{2k}\equiv i\;\mathrm{mod}\;rn$. The defining set *Z* of constacyclic C is the union of some $q^{2}$-cyclotomic cosets modulo rn. Constacyclic BCH codes have some properties that are similar to BCH codes. The following result gives the BCH bound of constacyclic codes.

**Proposition 2**  (The BCH bound for constacyclic codes [33,38,68])**.** *Assume that gcd(n, q)=1. Let C be a $q^{2}$-ary λ-constacyclic code of length n. If the generator polynomial g(x) of C has the elements $\left\{\eta^{1+ri}|0\leq i\leq d-2\right\}$ as the roots, where η is a primitive rn-th root of unity, then the minimum distance of C is at least d.*

From [33,37], we can see that the Hermitian dual $C^{{⊥}_{h}}$ of a λ-constacyclic code over $F_{q^{2}}$ is a $\lambda^{-q}$-constacyclic code. If C is a constacyclic code over $F_{q^{2}}$ with defining set Z, then the Hermitian dual $C^{{⊥}_{h}}$ has a defining set $Z^{{⊥}_{h}}=\left\{z\in O_{rn}|-qz\;\mathrm{mod}\;rn\notin Z\right\}.$ Moreover, it has the following result in [33,37].

**Lemma 1**  ([33,37])**.** *Let C be a $q^{2}$-ary constacyclic code of length n with defining set Z. Then C contains its Hermitian dual code if and only if Z∩−qZ=∅, where −qZ={−qzmodrn∣z∈Z}.*

### 3.2. Asymmetric QEC Codes

In this subsection, we present some definitions and basic results of binary asymmetric QEC codes, and then introduce the basic results of *q*-ary asymmetric QEC codes. Specific details can be found [51,53,57,65,71,72].

#### 3.2.1. Asymmetric QEC Codes for Qubit-Based Systems

In quantum information processing, a quantum bit (qubit) is a non-zero vector in the two-dimensional complex vector space $C^{2}$. Typically, a basis for $C^{2}$ is represented as |0〉 and |1〉, so a qubit can be represented as

(3) $$\alpha^{\prime }|0\rangle +\beta^{\prime }|1\rangle =\left(\begin{array}{c} \alpha^{\prime }\\ \beta^{\prime } \end{array}\right),$$

where $\alpha^{\prime },\beta^{\prime }\in C$ and $|\alpha^{\prime }{|}^{2}+{\left|\beta^{\prime }\right|}^{2}=1.$ A quantum state (*n*-qubit) is a non-zero vector in the tensor product space $C^{2^{n}}$, assuming the basis vector of the tensor product space is |a〉, where $|a\rangle =|a_{1}a_{2}\ldots a_{n}\rangle$ and $\left(a_{1},a_{2},\ldots ,a_{n}\right)\in F_{2}^{n}$. Therefore, a quantum state can be uniquely represented as $|v\rangle =\sum_{a\in F_{2}^{n}}c\left(a\right)|a\rangle$, where c(a) with $a\in F_{2}^{n}$ is a complex number that is not all zeros.

In quantum physics, a quantum state is a vector in $C^{2^{n}}$, and each quantum error is a unitary linear transformation in the complex vector space $C^{2^{n}}$. According to the CSS stabilizer theory, only independent error operators acting on each qubit need to be considered, and only three Pauli operators need to be considered for the error action on each qubit: $X=\left(\begin{array}{cc} 0 & 1\\ 1 & 0 \end{array}\right),$ $Z=\left(\begin{array}{cc} 1 & 0\\ 0 & -1 \end{array}\right)$ and $Y=\left(\begin{array}{cc} 0 & -i\\ i & 0 \end{array}\right).$ These three unitary operators act on the qubit $|v\rangle =\alpha^{\prime }|0\rangle +\beta^{\prime }|1\rangle$ as follows:

(4) $$X|v\rangle =\alpha^{\prime }|1\rangle +\beta^{\prime }|0\rangle ,$$

(5) $$Z|v\rangle =\alpha^{\prime }|0\rangle -\beta^{\prime }|1\rangle ,$$

and

(6) $$Y|v\rangle =i\alpha^{\prime }|1\rangle -i\beta^{\prime }|0\rangle ,$$

where $X^{2}=Y^{2}=Z^{2}=I_{2}$, Y=iXZ and $I_{2}=\left(\begin{array}{cc} 1 & 0\\ 0 & 1 \end{array}\right)$.

The form of each error operator on the complex space $C^{2^{n}}$ is $e=i^{\lambda }\omega_{1}⊗\omega_{2}⊗\ldots \omega_{n},$ where λ∈{0,1,2,3}, $\omega_{i}\in \left\{I_{2},X,Y,Z\right\}$ with 1≤i≤n, here $\omega =I_{2}$ indicates that the *i*-th qubit does not have an error. In fact, the Pauli operators and the phase factors {±1,±i} jointly constitute a Pauli group. For the basis vector $|a\rangle =|a_{1}a_{2}\ldots a_{n}\rangle$, it has

(7) $$e|a\rangle =i^{\lambda }\left(\omega_{1}|a_{1}\rangle \right)⊗\left(\omega_{2}|a_{2}\rangle \right)⊗\ldots ⊗\left(\omega_{n}|a_{n}\rangle \right).$$

In addition, for a *n*-qubit $|v\rangle =\sum_{a\in F_{2}^{n}}c\left(a\right)|a\rangle$, it has

(8) $$e|v\rangle =\sum_{a\in F_{2}^{n}}c\left(a\right)\left(e|a\rangle \right).$$

Let $G_{i}$ and $H_{i}$ be the parity check and generator matrices of a classical code $C_{i}$ with parameters $\left[n,k_{i},d_{i}\right]$ for i∈{1,2}. The stabilizer of a quantum code based on the parity check matrices $H_{1}$ and $H_{2}$ satisfies the following equation

$$H_{1}H_{2}^{T}+H_{2}H_{1}^{T}=0\left(mod\;2\right),$$

where $H_{i}^{T}$ denotes the transpose of the matrix $H_{i}$. The normalizer of the quantum code based on the generator matrices $G_{1}$ and $G_{2}$ satisfies the conditions $H_{1}G_{1}^{T}=0$ and $H_{2}G_{2}^{T}=0$.

Therefore, the CSS construction of a binary AQEC code can be stated as Definition 1, where one of the two classical codes controls phase-shift errors, while the other controls qubit-flip errors.

**Definition 1**  ([51])**.** *Given two classical codes $C_{1}$ and $C_{2}$ such that $C_{2}^{⊥}\subset C_{1}.$ If $G=\left(\begin{array}{cc} G_{1} & 0\\ 0 & G_{2} \end{array}\right)$, and $H=\left(\begin{array}{cc} H_{1} & 0\\ 0 & H_{2} \end{array}\right)$, then*

(9) $$H_{1}H_{2}^{T}+H_{2}H_{1}^{T}=0.$$

*Let $d_{x}=wt\left(C_{1}\backslash C_{2}^{⊥}\right)$ and $d_{z}=wt\left(C_{2}\backslash C_{1}^{⊥}\right)$, such that $d_{z}>d_{x}$ and $k_{1}+k_{2}>n$. Assuming that $C_{1}$ corrects the qubit-flip errors and $C_{2}$ corrects phase-shift errors, then there exist AQEC codes with parameters*

$${\left[\left[n,k_{1}+k_{2}-n,d_{z}/d_{x}\right]\right]}_{2}.$$

#### 3.2.2. Asymmetric QEC Codes for Qudit-Based Systems

Binary asymmetric QEC codes and *q*-ary asymmetric QEC codes are both designed to protect quantum information from noise and decoherence. The extension of binary asymmetric QEC codes to *q*-dimensional cases has practical significance, as in practical quantum computing, we may need to handle more qudits and may want to use different types of qudits to achieve more complex computations. Therefore, studying *q*-ary asymmetric QEC codes can help us better understand and design error-correcting schemes in practical quantum computing.

Let *H* be the Hilbert space $H=C^{q^{n}}=C^{q}⨂\ldots ⨂C^{q}.$ Let |x〉 be the vectors of an orthonormal basis of $C^{q}$, where the notions *x* are elements of $F_{q}.$ Consider $a,b\in F_{q},$ the unitary operators X(a) and Z(b) in $C^{q}$ are defined by

(10) X(a)|x〉=|x+a〉

and

(11) $$Z\left(b\right)|x\rangle =\omega^{tr(bx)}|x\rangle$$

respectively, where ω=exp(2πi/p) is a *p*-th root of unity and tr is the trace map from $F_{q}$ to $F_{p}$ (*p* is a prime). Consider that $a=\left(a_{1},a_{2},\ldots ,a_{n}\right)\in F_{q}^{n}$ and $b=\left(b_{1},b_{2},\ldots ,b_{n}\right)\in F_{q}^{n}.$ Let

(12) $$X\left(a\right)=X\left(a_{1}\right)⊗X\left(a_{2}\right)⊗\ldots ⊗X\left(a_{n}\right)$$

and

(13) $$Z\left(b\right)=Z\left(b_{1}\right)⊗Z\left(b_{2}\right)⊗\ldots ⊗Z\left(b_{n}\right)$$

be the tensor products of *n* error operators. The set

(14) $$E_{n}=\left\{X\left(a\right)Z\left(b\right)|a,b\in F_{q}^{n}\right\}$$

is an error basis on the complex vector space $C^{q^{n}}$ and the set

(15) $$G_{n}=\left\{\omega^{c}X\left(a\right)Z\left(b\right)|a,b\in F_{q}^{n},c\in F_{p}\right\}$$

is the error group associated with $E_{n}$. For a quantum error $e=\omega^{c}X\left(a\right)Z\left(b\right)\in G_{n}$, where the quantum weight $\omega_{Q}\left(e\right),$ the *X*-weight $\omega_{X}\left(e\right)$, and the *Z*-weight $\omega_{Z}\left(e\right)$ of *e* are defined, respectively, by

(16) $$\omega_{Q}\left(e\right)=♯\left\{i:1\leq i\leq n,\left(a_{i},b_{i}\right)\neq \left(0,0\right)\right\},$$

(17) $$\omega_{X}\left(e\right)=♯\left\{i:1\leq i\leq n,a_{i}\neq 0\right\},$$

(18) $$\omega_{Z}\left(e\right)=♯\left\{i:1\leq i\leq n,b_{i}\neq 0\right\}.$$

**Definition 2**  ([53])**.** *A q-ary asymmetric QEC code Q, denoted by ${\left[\left[n,k,d_{z}/d_{x}\right]\right]}_{q},$ is a $q^{k}$-dimensional subspace of the Hilbert space $C^{q^{n}}$, it can control all qudit-flip errors up to $\left\lfloor \left(d_{x}-1\right)/2\right\rfloor$ and all phase-shift errors up to $\left\lfloor \left(d_{z}-1\right)/2\right\rfloor$.*

### 3.3. Asymmetric EAQEC Codes

In this subsection, we present some definitions and some basic results about EAQEC codes in [45,46,48,50].

A *q*-ary ${\left[\left[n,k,d;c\right]\right]}_{q}$ EAQEC code can encode *k* information qudits into *n* channel qudits with the help of *c* pairs of maximally entangled states and correct up to $\left\lfloor \frac{d-1}{2}\right\rfloor$ errors, in which *d* is the minimum distance of the code. The performance of an entanglement-assisted quantum code can be assessed through the metric of net rate $\frac{k-c}{n}$. Let *H* be an (n−k)×n parity check matrix of C over $F_{q^{2}}.$ Then $C^{{⊥}_{h}}$ has an n×(n−k) generator matrix $H^{†}$, where $H^{†}$ is the conjugate transpose matrix of *H* over $F_{q^{2}}.$

**Definition 3**  (Asymmetric EAQEC codes [16,17,18])**.** *An ${\left[\left[n,k,d_{z}/d_{x};c\right]\right]}_{q^{2}}$ asymmetric EAQEC code Q over $F_{q^{2}}$ encodes k logical qudits into n physical qudits with the help of c copies of maximally entangled states, which can correct all qudit-flip errors up to $\left\lfloor \frac{d_{x}-1}{2}\right\rfloor$ and all phase-flip errors up to $\left\lfloor \frac{d_{z}-1}{2}\right\rfloor$.*

**Theorem 1**  ([16,73])**.** *Let $C_{1}$ and $C_{2}$ be two linear codes over $F_{q^{2}}$ with parameters ${\left[n,k_{i},d_{i}\right]}_{q^{2}}$ with the parity check matrices $H_{i}$, respectively, for i=1,2. Then there exists an asymmetric QEC code with parameters ${\left[\left[n,k_{1}+k_{2}-n+c,d_{z}/d_{x};c\right]\right]}_{q^{2}}$, where $d_{z}=wt\left(C_{1}\setminus \left(C_{1}\cap C_{2}^{{⊥}_{h}}\right)\right)$ and $d_{x}=wt\left(C_{2}\setminus \left(C_{2}\cap C_{1}^{{⊥}_{h}}\right)\right),$ with $d_{z}$ and $d_{x}$ as the minimum Hamming weight of the elements in the corresponding set, and*

(19) $$c=rank\left(H_{1}H_{2}^{†}\right)=dim\left(C_{1}^{{⊥}_{h}}\right)-dim\left(C_{1}^{{⊥}_{h}}\cap C_{2}\right)$$

 *is the number of required maximally entangled states.*

**Proposition 3**  (Quantum Singleton bound for asymmetric EAQEC codes [16,17])**.** *If an $[[n,k,d_{z}/d_{x};$ ${c]]}_{q^{2}}$ asymmetric EAQEC code C exists, then*

(20) $$d_{z}+d_{x}\leq n-k+2+c.$$

*If C satisfies the equality $d_{z}+d_{x}=n-k+2+c,$ then it is called an asymmetric EAQMDS code.*

## 4. Constructions of Asymmetric EAQMDS Codes

In [5,48,50,74], the authors defined the decomposition of the defining set of constacyclic codes, including cyclic codes and negacyclic codes. The following Definition 4 further extends the decomposition method for the defining set of constacyclic codes.

**Definition 4.**  *Let $C_{1}$ and $C_{2}$ be constacyclic codes of length n with defining sets $Z_{1}$ and $Z_{2}$, respectively, where gcd(n,q)=1. Assume that $Z_{11}=Z_{1}\cap \left(-qZ_{2}\right)$, $Z_{12}=Z_{1}\setminus Z_{11}$, and $Z_{21}=Z_{2}\cap \left(-qZ_{1}\right)$, $Z_{22}=Z_{2}\setminus Z_{21}$, where $-qZ_{1}=\left\{-qx|x\in Z_{1}\right\}$ and $-qZ_{2}=\left\{-qx|x\in Z_{2}\right\}$. Then $Z_{i}=Z_{i1}\cup Z_{i2}$ is called a decomposition of the defining set of $C_{i}$ with respect to $C_{[(i+1)\;mod\;2]+1}$ for i=1,2.*

**Lemma 2.**  *Let $Z_{1}$ and $Z_{2}$ be two defining sets of constacyclic code $C_{1}$ and $C_{2}$ with length n, respectively, where gcd(n,q)=1. Suppose that $Z_{i}=Z_{i1}\cup Z_{i2}$ is a decomposition of $Z_{i}$. Then the number of entangled states required is $c=|Z_{i1}|$ for i=1,2.*

**Proof.**  Assume that the defining sets $Z_{1}$ and $Z_{2}$ can generate constacyclic codes $C_{1}$ and $C_{2}$, respectively. Let the parity check matrices of $C_{1}$ and $C_{2}$ over $F_{q^{2}}$ be $H_{1}$ and $H_{2}$, respectively. Therefore,

(21) $$H_{1}=\left(\begin{array}{c} H_{11}\\ H_{12} \end{array}\right),$$

and

(22) $$H_{2}=\left(\begin{array}{c} H_{21}\\ H_{22} \end{array}\right),$$

where $H_{11}$ and $H_{12}$ are parity check matrices of constacyclic codes generated by defining sets with $Z_{11}$ and $Z_{12}$, respectively, and $H_{21}$ and $H_{22}$ are parity check matrices of constacyclic codes generated by defining sets with $Z_{21}$ and $Z_{22}$, respectively.

Hence,

(23) $$H_{1}H_{2}^{†}=\left(\begin{array}{cc} H_{11}H_{21}^{†} & H_{11}H_{22}^{†}\\ H_{12}H_{21}^{†} & H_{12}H_{22}^{†} \end{array}\right).$$

From Definition 4, it has

(24) $$H_{1}H_{2}^{†}=\left(\begin{array}{cc} H_{11}H_{21}^{†} & 0\\ 0 & 0 \end{array}\right).$$

Hence, it has $c=rank\left(H_{1}H_{2}^{†}\right)=rank\left(H_{11}H_{21}^{†}\right)=\left|Z_{i1}\right|$. □

From Lemma 2, if $C_{1}=C_{2}$, then the following Corollary 1 can be derived easily.

**Corollary 1.**  *Let Z be a defining set of constacyclic code C with length n, where gcd(n,q)=1. Suppose that $Z=Z_{1}\cup Z_{2}$ is a decomposition of Z. Then the number of entangled states required is $c=\;|Z_{1}|$, where $Z_{1}=Z\cap \left(-qZ\right)$, $Z_{2}=Z\setminus Z_{1}$, and −qZ={−qx|x∈Z}.*

**Lemma 3**  ([11])**.** *Let $n=\frac{q^{2}+1}{\beta }$ and $s=\frac{q^{2}+1}{2}$, where q is an odd prime power of the form 2βm+β+t or 2βm+β−t, m is a positive integer, and t≥2 is even, such that $\beta =t^{2}+1$. Then $C_{s}=\left\{s\right\}$, $C_{s+\frac{q+1}{2}n}=\left\{s+\frac{q+1}{2}n\right\}$ and $C_{s-(q+1)i}=\left\{s-\left(q+1\right)i,s+\left(q+1\right)i\right\}$ for $1\leq i\leq \frac{n}{2}-1$.*

**Lemma 4.**  *Let $n=\frac{q^{2}+1}{\beta }$, $s=\frac{q^{2}+1}{2}$ and r=q+1, where q is an odd prime power of the form 2βm+β+t, m is a positive integer, and t≥2 is even, such that $\beta =t^{2}+1$. If $C_{1}$ and $C_{2}$ are two $q^{2}$-ary constacyclic codes whose defining sets are given by $Z_{1}=\cup_{i=0}^{\delta_{1}}C_{s-(q+1)i}$ and $Z_{2}=\cup_{i=0}^{\delta_{2}}C_{s-(q+1)i}$, respectively, where $0\leq \delta_{1}\leq \frac{q-\beta -t}{2\beta }$ and $\frac{q+\beta -t}{2\beta }\leq \delta_{2}\leq \frac{q^{2}+1}{2\beta }-q\delta_{1}-1$, then $C_{1}^{{⊥}_{h}}\subseteq C_{2}$.*

**Proof.**  If $C_{1}$ and $C_{2}$ are two $q^{2}$-ary constacyclic codes whose defining sets are given by $Z_{1}=\cup_{i=0}^{\delta_{1}}C_{s-(q+1)i}$ and $Z_{2}=\cup_{i=0}^{\delta_{2}}C_{s-(q+1)i}$ from Lemma 3, respectively, where $0\leq \delta_{1}\leq \frac{q-\beta -t}{2\beta }$ and $\frac{q+\beta -t}{2\beta }\leq \delta_{2}\leq \frac{q^{2}+1}{2\beta }-q\delta_{1}-1$, then $C_{1}$ and $C_{2}$ are two MDS constacyclic codes with parameters ${\left[n,n-2\delta_{1}-1,2\delta_{1}+2\right]}_{q^{2}}$ and ${\left[n,n-2\delta_{2}-1,2\delta_{2}+2\right]}_{q^{2}}$, respectively, from Propositions 1 and 2. For $0\leq \delta_{1}\leq \frac{q-\beta -t}{2\beta }$ and $\frac{q+\beta -t}{2\beta }\leq \delta_{2}\leq \frac{q^{2}+1}{2\beta }-q\delta_{1}-1$, it has $C_{1}^{{⊥}_{h}}\subseteq C_{2}$. In fact, it only needs to show that $Z_{2}\cap -qZ_{1}=\emptyset$. If $Z_{2}\cap -qZ_{1}\neq \emptyset$, then there exist two integers $\delta_{1}^{\prime }$ and $\delta_{2}^{\prime }$ such that $s-r\delta_{2}^{\prime }\equiv -q\left(s-r\delta_{1}^{\prime }\right)q^{2k}\;mod\;rn$ for k∈{0,1}, where $0\leq \delta_{1}^{\prime }\leq \frac{q-\beta -t}{2\beta }$ and $\frac{q+\beta -t}{2\beta }\leq \delta_{2}^{\prime }\leq \frac{q^{2}+1}{2\beta }-q\delta_{1}^{\prime }-1$.

If k=0, it has $s-r\delta_{2}^{\prime }\equiv -q\left(s-r\delta_{1}^{\prime }\right)\equiv -qs+qr\delta_{1}^{\prime }\;mod\;rn$, i.e., $s\equiv \delta_{2}^{\prime }+q\delta_{1}^{\prime }\;mod\;n$. Since $\frac{q+\beta -t}{2\beta }\leq \delta_{2}^{\prime }+q\delta_{1}^{\prime }\leq \frac{q^{2}+1}{2\beta }-1$, which is in contradiction with $s\equiv \delta_{2}^{\prime }+q\delta_{1}^{\prime }\;mod\;n$.

If k=1, it has $s-r\delta_{2}^{\prime }\equiv -\left(s-r\delta_{1}^{\prime }\right)q^{3}\equiv -qs+rq^{3}\delta_{1}^{\prime }\;mod\;rn$, i.e., $s\equiv \delta_{2}^{\prime }-q\delta_{1}^{\prime }\;mod\;n$. If $\delta_{1}^{\prime }=0$, then it has $\delta_{2}^{\prime }\equiv s\;mod\;n$ that is in contradiction with $\frac{q+\beta -t}{2\beta }\leq \delta_{2}^{\prime }\leq \frac{q^{2}+1}{2\beta }-q\delta_{1}^{\prime }-1$. Now we consider that $s+q\delta_{1}^{\prime }\equiv \delta_{2}^{\prime }\;mod\;n$ for $1\leq \delta_{1}^{\prime }\leq \frac{q-\beta -t}{2\beta }$ and $\frac{q+\beta -t}{2\beta }\leq \delta_{2}^{\prime }\leq \frac{q^{2}+1}{2\beta }-q\delta_{1}^{\prime }-1$. Since $s+q\delta_{1}^{\prime }\equiv \delta_{2}^{\prime }\;mod\;n$ is equivalent to $\frac{q^{2}+1}{2\beta }+q\delta_{1}^{\prime }\equiv \delta_{2}^{\prime }\;mod\;n$, we have $\frac{q^{2}+1}{2\beta }+q\leq \frac{q^{2}+1}{2\beta }+q\delta_{1}^{\prime }\leq \frac{q^{2}+1}{2\beta }+q\left(\frac{q-\beta -t}{2\beta }\right)=\frac{2q^{2}-\left(\beta +t\right)q+1}{2\beta }$, which is in contradiction with $\frac{q+\beta -t}{2\beta }\leq \delta_{2}^{\prime }\leq \frac{q^{2}+1}{2\beta }-q\delta_{1}^{\prime }-1\leq \frac{q^{2}+1}{2\beta }-q-1$. Therefore, it has $C_{1}^{{⊥}_{h}}\subseteq C_{2}$. □

**Theorem 2.**  *Let $n=\frac{q^{2}+1}{\beta }$ and $s=\frac{q^{2}+1}{2}$, where q=2βm+β+t is an odd prime power with a positive integer m, and t≥2 is even, such that $\beta =t^{2}+1$. For $0\leq \delta_{1}\leq \frac{q-\beta -t}{2\beta }$ and $\frac{q+\beta -t}{2\beta }\leq \delta_{2}\leq \frac{q^{2}+1}{2\beta }-q\delta_{1}+vq-1$, then there exist asymmetric EAQMDS codes with parameters ${\left[\left[n,n-2\left(\delta_{1}+\delta_{2}+1-v\right),2\delta_{2}+2/2\delta_{1}+2;2v\right]\right]}_{q^{2}}$, where $0\leq v\leq \delta_{1}$.*

**Proof.**  If $C_{1}$ and $C_{2}$ are two $q^{2}$-ary constacyclic codes whose defining sets are given by $Z_{1}=\cup_{i=0}^{\delta_{1}}C_{s-(q+1)i}$ and $Z_{2}=\cup_{i=0}^{\delta_{2}}C_{s-(q+1)i}$, respectively, where $0\leq \delta_{1}\leq \frac{q-\beta -t}{2\beta }$ and $\frac{q+\beta -t}{2\beta }\leq \delta_{2}\leq \frac{q^{2}+1}{2\beta }-q\delta_{1}+vq-1$, then $C_{1}$ and $C_{2}$ are two MDS constacyclic codes with parameters ${\left[n,n-2\delta_{1}-1,2\delta_{1}+2\right]}_{q^{2}}$ and ${\left[n,n-2\delta_{2}-1,2\delta_{2}+2\right]}_{q^{2}}$, respectively, from Propositions 1 and 2. From Lemma 4, it has that $C_{1}^{{⊥}_{h}}\subseteq C_{2}$ for $0\leq \delta_{1}\leq \frac{q-\beta -t}{2\beta }$ and $\frac{q+\beta -t}{2\beta }\leq \delta_{2}\leq \frac{q^{2}+1}{2\beta }-q\delta_{1}-1$, which implies that v=0. At this point, the asymmetric EAQMDS codes degenerate into asymmetric quantum MDS codes.

Now, we will discuss the case $1\leq v\leq \delta_{1}$. The range $\delta_{2}$ can be divided into some intervals with $I_{0}=\left[\frac{q+\beta -t}{2\beta },\frac{q^{2}+1}{2\beta }-q\delta_{1}-1\right],$ $I_{1}=\left[\frac{q^{2}+1}{2\beta }-q\delta_{1},\frac{q^{2}+1}{2\beta }-q\delta_{1}+q-1\right],$ $I_{2}=\left[\frac{q^{2}+1}{2\beta }-q\delta_{1}+q,\frac{q^{2}+1}{2\beta }-q\delta_{1}+2q-1\right]$, $I_{3}=\left[\frac{q^{2}+1}{2\beta }-q\delta_{1}+2q,\frac{q^{2}+1}{2\beta }-q\delta_{1}+3q-1\right],$*…*, $I_{v}=\left[\frac{q^{2}+1}{2\beta }-q\delta_{1}+\left(v-1\right)q,\frac{q^{2}+1}{2\beta }-q\delta_{1}+vq-1\right],$ where $1\leq v\leq \delta_{1}$. From Lemma 2, it only needs to show that $|Z_{11}\left|\;=\;\right|Z_{1}\cap \left(-qZ_{2}\right)|\;=2v$ (or $|Z_{21}\left|\;=\;\right|Z_{2}\cap \left(-qZ_{1}\right)|\;=2v$).

Since

(25) $$\begin{array}{cc} \; & -qZ_{1}\cap Z_{2}\\ \; & =-q\left(\cup_{i=0}^{\delta_{1}}C_{s-(q+1)i}\right)\cap \left(\cup_{i=0}^{\delta_{2}}C_{s-(q+1)i}\right)\\ \; & =-q\left(\cup_{i=0}^{\delta_{1}}C_{s-(q+1)i}\right)\cap \left(\cup_{l=0}^{v}\cup_{j\in I_{l}}C_{s-(q+1)j}\right)\\ \; & =-q\left(\cup_{i=0}^{\delta_{1}}C_{s-(q+1)i}\right)\cap \left(\cup_{j\in I_{0}}C_{s-(q+1)j}\cup \left(\cup_{j\in I_{1}}C_{s-(q+1)j}\right)\cup \ldots \cup \left(\cup_{j\in I_{v}}C_{s-(q+1)j}\right)\right)\\ \; & =\left(-q\cup_{i=0}^{\delta_{1}}C_{s-(q+1)i}\cap \left(\cup_{j\in I_{0}}C_{s-(q+1)j}\right)\right)\cup \left(-q\cup_{i=0}^{\delta_{1}}C_{s-(q+1)i}\cap \left(\cup_{j\in I_{1}}C_{s-(q+1)j}\right)\right)\cup \\ \; & \ldots \cup (-q\cup_{i=0}^{\delta_{1}}C_{s-(q+1)i}\cap \left(\cup_{j\in I_{v}}C_{s-(q+1)j}\right)) \end{array}$$

Here, we will discuss the above equation specifically for different cases, as follows:

(1) If v=1, then $I_{1}=\left[\frac{q^{2}+1}{2\beta }-q\delta_{1},\frac{q^{2}+1}{2\beta }-q\delta_{1}+q-1\right],$

(26) $$\begin{array}{cc} \; & -q(s-\left(q+1\right)\left(\frac{q^{2}+1}{2\beta }-q\delta_{1}\right))\\ \; & \equiv -\left(q+1\right)s+s+q\left(q+1\right)\frac{q^{2}+1}{2\beta }-q^{2}\left(q+1\right)\delta_{1}\\ \; & \equiv s-\left(q+1\right)s+\left(q^{2}-1+q+1\right)\frac{q^{2}+1}{2\beta }-\left(q^{2}+1-1\right)\left(q+1\right)\delta_{1}\\ \; & \equiv s-\left(q+1\right)s+\left(q+1\right)\frac{q^{2}+1}{2\beta }+\left(q+1\right)\delta_{1}\\ \; & \equiv s-\left(q+1\right)\left(\frac{q^{2}+1}{2}-\frac{q^{2}+1}{2\beta }-\delta_{1}\right)\\ \; & \equiv s-\left(q+1\right)\left(\frac{\left(q^{2}+1\right)t^{2}}{2\beta }-\delta_{1}\right)\\ \; & \equiv s+\left(q+1\right)\delta_{1}mod\left(q+1\right)n, \end{array}$$

and

(27) $$\begin{array}{cc} \; & -q(s+\left(q+1\right)\left(\frac{q^{2}+1}{2\beta }-q\delta_{1}\right))\\ & \equiv -\left(q+1\right)s+s-q\left(q+1\right)\frac{q^{2}+1}{2\beta }+q^{2}\left(q+1\right)\delta_{1}\\ \; & \equiv s-\left(q+1\right)s-\left(q^{2}-1+q+1\right)\frac{q^{2}+1}{2\beta }+\left(q^{2}+1-1\right)\left(q+1\right)\delta_{1}\\ \; & \equiv s-\left(q+1\right)s-\left(q+1\right)\frac{q^{2}+1}{2\beta }-\left(q+1\right)\delta_{1}\\ \; & \equiv s-\left(q+1\right)\left(\frac{q^{2}+1}{2}+\frac{q^{2}+1}{2\beta }+\delta_{1}\right)\\ \; & \equiv s-\left(q+1\right)\left(\frac{\left(q^{2}+1\right)\left(\beta +1\right)}{2\beta }+\delta_{1}\right)\\ \; & \equiv s-\left(q+1\right)\delta_{1}mod\left(q+1\right)n, \end{array}$$

which implies $-qC_{s-\left(q+1\right)\left(\frac{q^{2}+1}{2\beta }-q\delta_{1}\right)}=C_{s-\left(q+1\right)\delta_{1}}$.

Moreover,

(28) $$\begin{array}{cc} \; & -q\left(\cup_{i=0}^{\delta_{1}}C_{s-(q+1)i}\right)\cap \left(\cup_{j\in I_{1}}C_{s-(q+1)j}\right)\\ \; & =-q\left(\cup_{i=0}^{\delta_{1}}C_{s-(q+1)i}\right)\cap \left(\cup_{j=\frac{q^{2}+1}{2\beta }-q\delta_{1}+1}^{\frac{q^{2}+1}{2\beta }-q\delta_{1}+q-1}C_{s-(q+1)j}\cup C_{s-\left(q+1\right)\left(\frac{q^{2}+1}{2\beta }-q\delta_{1}\right)}\right)\\ \; & =(-q\left(\cup_{i=0}^{\delta_{1}}C_{s-(q+1)i}\right)\cap \left(\cup_{j=\frac{q^{2}+1}{2\beta }-q\delta_{1}+1}^{\frac{q^{2}+1}{2\beta }-q\delta_{1}+q-1}C_{s-(q+1)j}\right))\\ \; & \cup (-q\left(\cup_{i=0}^{\delta_{1}}C_{s-(q+1)i}\right)\cap C_{s-\left(q+1\right)\left(\frac{q^{2}+1}{2\beta }-q\delta_{1}\right)}). \end{array}$$

From Lemma 4, we can see that

(29) $$-q\left(\cup_{i=0}^{\delta_{1}}C_{s-(q+1)i}\right)\cap C_{s-\left(q+1\right)\left(\frac{q^{2}+1}{2\beta }-q\delta_{1}\right)}=C_{s-\left(q+1\right)\left(\frac{q^{2}+1}{2\beta }-q\delta_{1}\right)}.$$

Hence, Formula (28) is equivalent to

(30) $$\begin{array}{cc} \; & -q\left(\cup_{i=0}^{\delta_{1}}C_{s-(q+1)i}\right)\cap \left(\cup_{j\in I_{1}}C_{s-(q+1)j}\right)\\ \; & =-q\left(\cup_{i=0}^{\delta_{1}}C_{s-(q+1)i}\right)\cap \left(\cup_{j=\frac{q^{2}+1}{2\beta }-q\delta_{1}+1}^{\frac{q^{2}+1}{2\beta }-q\delta_{1}+q-1}C_{s-(q+1)j}\right)\cup C_{s-\left(q+1\right)\left(\frac{q^{2}+1}{2\beta }-q\delta_{1}\right)}. \end{array}$$

Next, we will prove that if $-q\left(\cup_{i=0}^{\delta_{1}}C_{s-(q+1)i}\right)\cap \left(\cup_{j=\frac{q^{2}+1}{2\beta }-q\delta_{1}+1}^{\frac{q^{2}+1}{2\beta }-q\delta_{1}+q-1}C_{s-(q+1)j}\right)=\emptyset$ holds, then the equation

(31) $$\begin{array}{c} -q\left(\cup_{i=0}^{\delta_{1}}C_{s-(q+1)i}\right)\cap \left(\cup_{j\in I_{1}}C_{s-(q+1)j}\right)=C_{s-\left(q+1\right)\left(\frac{q^{2}+1}{2\beta }-q\delta_{1}\right)} \end{array}$$

is true.

Assume that $-q\left(\cup_{i=0}^{\delta_{1}}C_{s-(q+1)i}\right)\cap \left(\cup_{j=\frac{q^{2}+1}{2\beta }-q\delta_{1}+1}^{\frac{q^{2}+1}{2\beta }-q\delta_{1}+q-1}C_{s-(q+1)j}\right)\neq \emptyset$, then there exist two integers, $\delta_{1}^{\prime }$ and $\delta_{2}^{\prime }$, such that $s-\left(q+1\right)\delta_{2}^{\prime }\equiv -q\left(s-\left(q+1\right)\delta_{1}^{\prime }\right)q^{2k}\;mod\;\left(q+1\right)n$ for k∈{0,1}, where $0\leq \delta_{1}^{\prime }\leq \frac{q-\beta -t}{2\beta }$ and $\frac{q^{2}+1}{2\beta }-q\delta_{1}^{\prime }+1\leq \delta_{2}^{\prime }\leq \frac{q^{2}+1}{2\beta }-q\delta_{1}^{\prime }+q-1$.

If k=0, it has $s-\left(q+1\right)\delta_{2}^{\prime }\equiv -q\left(s-\left(q+1\right)\delta_{1}^{\prime }\right)\equiv -qs+q\left(q+1\right)\delta_{1}^{\prime }\;mod\;\left(q+1\right)n$, i.e., $s\equiv \delta_{2}^{\prime }+q\delta_{1}^{\prime }\;mod\;n$. Since $\frac{q^{2}+1}{2\beta }+1\leq \delta_{2}^{\prime }+q\delta_{1}^{\prime }\leq \frac{q^{2}+1}{2\beta }+q-1$, which is in contradiction with $s\equiv \delta_{2}^{\prime }+q\delta_{1}^{\prime }\;mod\;n$.

If k=1, it has $s-\left(q+1\right)\delta_{2}^{\prime }\equiv -\left(s-\left(q+1\right)\delta_{1}^{\prime }\right)q^{3}\equiv -qs+\left(q+1\right)q^{3}\delta_{1}^{\prime }\;mod\;\left(q+1\right)n$, i.e., $s\equiv \delta_{2}^{\prime }-q\delta_{1}^{\prime }\;mod\;n$. If $\delta_{1}^{\prime }=0$, then it has $\delta_{2}^{\prime }\equiv s\;mod\;n$ that is in contradiction with $\frac{q^{2}+1}{2\beta }+1\leq \delta_{2}^{\prime }\leq \frac{q^{2}+1}{2\beta }+q-1$. Now we consider that $s+q\delta_{1}^{\prime }\equiv \delta_{2}^{\prime }\;mod\;n$ for $1\leq \delta_{1}^{\prime }\leq \frac{q-\beta -t}{2\beta }$ and $\frac{q^{2}+1}{2\beta }-q\delta_{1}^{\prime }+1\leq \delta_{2}^{\prime }\leq \frac{q^{2}+1}{2\beta }-q\delta_{1}^{\prime }+q-1$. Since $s+q\delta_{1}^{\prime }\equiv \delta_{2}^{\prime }\;mod\;n$ is equivalent to $q\delta_{1}^{\prime }+\frac{q^{2}+1}{2\beta }\equiv \delta_{2}^{\prime }\;mod\;n$, it has $\frac{q^{2}+1}{2\beta }+q\leq \frac{q^{2}+1}{2\beta }+q\delta_{1}^{\prime }\leq \frac{q^{2}+1}{2\beta }+q\left(\frac{q-\beta -t}{2\beta }\right)=\frac{2q^{2}-\left(\beta +t\right)q+1}{2\beta }$, which is in contradiction with $\frac{q^{2}+1}{2\beta }-q\delta_{1}^{\prime }+1\leq \delta_{2}^{\prime }\leq \frac{q^{2}+1}{2\beta }-q\delta_{1}^{\prime }+q-1$. Therefore, it has $-q\left(\cup_{i=0}^{\delta_{1}}C_{s-(q+1)i}\right)\cap \left(\cup_{i=\frac{q^{2}+1}{2\beta }-q\delta_{1}+1}^{\frac{q^{2}+1}{2\beta }-q\delta_{1}+q-1}C_{s-(q+1)i}\right)=\emptyset$.

From the above discussion, we can see that Formula (31) holds. Hence, the number of entangled states required is 2 when v=1.

(2) Here, we prove the case for $I_{v}=\left[\frac{q^{2}+1}{2\beta }-q\delta_{1}+\left(v-1\right)q,\frac{q^{2}+1}{2\beta }-q\delta_{1}+vq-1\right]$, and the same way can be proven for other cases as well. If $I_{v}=\left[\frac{q^{2}+1}{2\beta }-q\delta_{1}+\left(v-1\right)q,\frac{q^{2}+1}{2\beta }-q\delta_{1}+vq-1\right],$ then

(32) $$\begin{array}{cc} \; & -q(s-\left(q+1\right)\left(\frac{q^{2}+1}{2\beta }-q\delta_{1}+q\left(v-1\right)\right))\\ & \equiv -\left(q+1\right)s+s+q\left(q+1\right)\frac{q^{2}+1}{2\beta }-q^{2}\left(q+1\right)\left(\delta_{1}-v+1\right)\\ \; & \equiv s-\left(q+1\right)s+\left(q^{2}-1+q+1\right)\frac{q^{2}+1}{2\beta }-\left(q^{2}+1-1\right)\left(q+1\right)\left(\delta_{1}-v+1\right)\\ \; & \equiv s-\left(q+1\right)s+\left(q+1\right)\frac{q^{2}+1}{2\beta }+\left(q+1\right)\left(\delta_{1}-v+1\right)\\ \; & \equiv s-\left(q+1\right)\left(\frac{q^{2}+1}{2}-\frac{q^{2}+1}{2\beta }-\delta_{1}+v-1\right)\\ \; & \equiv s-\left(q+1\right)\left(\frac{\left(q^{2}+1\right)t^{2}}{2\beta }-\delta_{1}+v-1\right)\\ \; & \equiv s+\left(q+1\right)\left(\delta_{1}-v+1\right)mod\left(q+1\right)n, \end{array}$$

and

(33) $$\begin{array}{cc} \; & -q(s+\left(q+1\right)\left(\frac{q^{2}+1}{2\beta }-q\delta_{1}+q\left(v-1\right)\right))\\ \; & \equiv -\left(q+1\right)s+s-q\left(q+1\right)\frac{q^{2}+1}{2\beta }+q^{2}\left(q+1\right)\left(\delta_{1}-v+1\right)\\ \; & \equiv s-\left(q+1\right)s-\left(q^{2}-1+q+1\right)\frac{q^{2}+1}{2\beta }+\left(q^{2}+1-1\right)\left(q+1\right)\left(\delta_{1}-v+1\right)\\ \; & \equiv s-\left(q+1\right)s-\left(q+1\right)\frac{q^{2}+1}{2\beta }-\left(q+1\right)\left(\delta_{1}-v+1\right)\\ \; & \equiv s-\left(q+1\right)\left(\frac{q^{2}+1}{2}+\frac{q^{2}+1}{2\beta }+\delta_{1}-v+1\right)\\ \; & \equiv s-\left(q+1\right)\left(\frac{\left(q^{2}+1\right)\left(\beta +1\right)}{2\beta }+\delta_{1}-v+1\right)\\ \; & \equiv s-\left(q+1\right)\left(\delta_{1}-v+1\right)mod\left(q+1\right)n, \end{array}$$

which implies $-qC_{s-\left(q+1\right)(\frac{q^{2}+1}{2\beta }-q\delta_{1}+q\left(v-1\right))}=C_{s-\left(q+1\right)\left(\delta_{1}-v+1\right)}$.

Moreover,

(34) $$\begin{array}{cc} \; & -q\cup_{i=0}^{\delta_{1}}C_{s-(q+1)i}\cap \left(\cup_{j\in I_{v}}C_{s-(q+1)j}\right)\\ \; & =-q\left(\cup_{i=0}^{\delta_{1}}C_{s-(q+1)i}\right)\cap \left(\cup_{j=\frac{q^{2}+1}{2\beta }-q\delta_{1}+\left(v-1\right)q}^{\frac{q^{2}+1}{2\beta }-q\delta_{1}+vq-1}C_{s-(q+1)j}\right)\\ \; & =-q\left(\cup_{i=0}^{\delta_{1}}C_{s-(q+1)i}\right)\cap \left(\cup_{j=\frac{q^{2}+1}{2\beta }-q\delta_{1}+\left(v-1\right)q+1}^{\frac{q^{2}+1}{2\beta }-q\delta_{1}+vq-1}C_{s-(q+1)j}\cup C_{s-\left(q+1\right)(\frac{q^{2}+1}{2\beta }-q\delta_{1}+\left(v-1\right)q)}\right)\\ \; & =(-q\left(\cup_{i=0}^{\delta_{1}}C_{s-(q+1)i}\right)\cap \left(\cup_{j=\frac{q^{2}+1}{2\beta }-q\delta_{1}+\left(v-1\right)q+1}^{\frac{q^{2}+1}{2\beta }-q\delta_{1}+vq-1}C_{s-(q+1)j}\right))\\ \; & \cup (-q\left(\cup_{i=0}^{\delta_{1}}C_{s-(q+1)i}\right)\cap C_{s-\left(q+1\right)(\frac{q^{2}+1}{2\beta }-q\delta_{1}+q\left(v-1\right))}). \end{array}$$

From Lemma 4, we can see that

(35) $$-q\left(\cup_{i=0}^{\delta_{1}}C_{s-(q+1)i}\right)\cap C_{s-\left(q+1\right)(\frac{q^{2}+1}{2\beta }-q\delta_{1}+q\left(v-1\right))}=C_{s-\left(q+1\right)(\frac{q^{2}+1}{2\beta }-q\delta_{1}+q\left(v-1\right))}.$$

Hence, Formula (34) is equivalent to

(36) $$\begin{array}{cc} \; & -q\left(\cup_{i=0}^{\delta_{1}}C_{s-(q+1)i}\right)\cap \left(\cup_{j\in I_{v}}C_{s-(q+1)j}\right)\\ \; & =-q\left(\cup_{i=0}^{\delta_{1}}C_{s-(q+1)i}\right)\cap \left(\cup_{j=\frac{q^{2}+1}{2\beta }-q\delta_{1}+\left(v-1\right)q+1}^{\frac{q^{2}+1}{2\beta }-q\delta_{1}+vq-1}C_{s-(q+1)j}\right)\cup C_{s-\left(q+1\right)(\frac{q^{2}+1}{2\beta }-q\delta_{1}+q\left(v-1\right))}. \end{array}$$

Next, we will prove that if $-q\left(\cup_{i=0}^{\delta_{1}}C_{s-(q+1)i}\right)\cap \left(\cup_{j=\frac{q^{2}+1}{2\beta }-q\delta_{1}+\left(v-1\right)q+1}^{\frac{q^{2}+1}{2\beta }-q\delta_{1}+vq-1}C_{s-(q+1)j}\right)=\emptyset$ holds, then the equation

(37) $$\begin{array}{c} -q\left(\cup_{i=0}^{\delta_{1}}C_{s-(q+1)i}\right)\cap \left(\cup_{j\in I_{v}}C_{s-(q+1)j}\right)=C_{s-\left(q+1\right)(\frac{q^{2}+1}{2\beta }-q\delta_{1}+q\left(v-1\right))} \end{array}$$

is true.

Assume that $-q\left(\cup_{i=0}^{\delta_{1}}C_{s-(q+1)i}\right)\cap \left(\cup_{j=\frac{q^{2}+1}{2\beta }-q\delta_{1}+\left(v-1\right)q+1}^{\frac{q^{2}+1}{2\beta }-q\delta_{1}+vq-1}C_{s-(q+1)j}\right)\neq \emptyset$, then there exist two integers $\delta_{1}^{\prime }$ and $\delta_{2}^{\prime }$ such that $s-\left(q+1\right)\delta_{2}^{\prime }\equiv -q\left(s-\left(q+1\right)\delta_{1}^{\prime }\right)q^{2k}\;mod\;\left(q+1\right)n$ for k∈{0,1}, where $0\leq \delta_{1}^{\prime }\leq \frac{q-\beta -t}{2\beta }$ and $\frac{q^{2}+1}{2\beta }-q\delta_{1}^{\prime }+\left(v-1\right)q+1\leq \delta_{2}^{\prime }\leq \frac{q^{2}+1}{2\beta }-q\delta_{1}^{\prime }+vq-1$.

If k=0, it has $s-\left(q+1\right)\delta_{2}^{\prime }\equiv -q\left(s-\left(q+1\right)\delta_{1}^{\prime }\right)\equiv -qs+q\left(q+1\right)\delta_{1}^{\prime }\;mod\;\left(q+1\right)n$, i.e., $s\equiv \delta_{2}^{\prime }+q\delta_{1}^{\prime }\;mod\;n$. Since $\frac{q^{2}+1}{2\beta }+\left(v-1\right)q+1\leq \delta_{2}^{\prime }+q\delta_{1}^{\prime }\leq \frac{q^{2}+1}{2\beta }+vq-1$, which is in contradiction with $s\equiv \delta_{2}^{\prime }+q\delta_{1}^{\prime }\;mod\;n$.

If k=1, it has $s-\left(q+1\right)\delta_{2}^{\prime }\equiv -\left(s-\left(q+1\right)\delta_{1}^{\prime }\right)q^{3}\equiv -qs+\left(q+1\right)q^{3}\delta_{1}^{\prime }\;mod\;\left(q+1\right)n$, i.e., $s\equiv \delta_{2}^{\prime }-q\delta_{1}^{\prime }\;mod\;n$. If $\delta_{1}^{\prime }=0$, then it has $\delta_{2}^{\prime }\equiv s\;mod\;n$ that is in contradiction with $\frac{q^{2}+1}{2\beta }+\left(v-1\right)q+1\leq \delta_{2}^{\prime }\leq \frac{q^{2}+1}{2\beta }+vq-1$. Now we consider that $s+q\delta_{1}^{\prime }\equiv \delta_{2}^{\prime }\;mod\;n$ for $1\leq \delta_{1}^{\prime }\leq \frac{q-\beta -t}{2\beta }$ and $\frac{q^{2}+1}{2\beta }-q\delta_{1}^{\prime }+\left(v-1\right)q+1\leq \delta_{2}^{\prime }\leq \frac{q^{2}+1}{2\beta }-q\delta_{1}^{\prime }+vq-1$. Since $s+q\delta_{1}^{\prime }\equiv \delta_{2}^{\prime }\;mod\;n$ is equivalent to $\frac{q^{2}+1}{2\beta }+q\delta_{1}^{\prime }\equiv \delta_{2}^{\prime }\;mod\;n$, it has $\frac{q^{2}+1}{2\beta }+q\leq \frac{q^{2}+1}{2\beta }+q\delta_{1}^{\prime }\leq \frac{q^{2}+1}{2\beta }+q\left(\frac{q-\beta -t}{2\beta }\right)=\frac{2q^{2}-\left(\beta +t\right)q+1}{2\beta }$, which is in contradiction with $\frac{q^{2}+1}{2\beta }-q\delta_{1}^{\prime }+\left(v-1\right)q+1\leq \delta_{2}^{\prime }\leq \frac{q^{2}+1}{2\beta }-q\delta_{1}^{\prime }+vq-1$. Therefore, it has $-q\left(\cup_{i=0}^{\delta_{1}}C_{s-(q+1)i}\right)\cap \left(\cup_{j=\frac{q^{2}+1}{2\beta }-q\delta_{1}+\left(v-1\right)q+1}^{\frac{q^{2}+1}{2\beta }-q\delta_{1}+vq-1}C_{s-(q+1)j}\right)=\emptyset .$

According to the above discussion, the number of entangled states is 2v, where $0\leq v\leq \delta_{1}$. Therefore, we can see that asymmetric EAQMDS codes with parameters ${\left[\left[n,n-2\left(\delta_{1}+\delta_{2}+1-v\right),2\delta_{2}+2/2\delta_{1}+2;2v\right]\right]}_{q^{2}}$ exist from Theorem 1 and Proposition 3. □

**Example 1.**  *Let t=2 and q=17, then n=58, $0\leq \delta_{1}\leq 1$, $2\leq \delta_{2}\leq 29-17\delta_{1}+17v-1$ and $0\leq v\leq \delta_{1}$. Some asymmetric EAQMDS codes with c=2 derived from Theorem 2 are listed in Table 1.*

Similar to Lemma 4 and Theorem 2, we can also obtain the following Lemma 5 and Theorem 3.

**Lemma 5.**  *Let $n=\frac{q^{2}+1}{\beta }$ and $s=\frac{q^{2}+1}{2}$, where q is an odd prime power of the form 2βm+β−t, m is a positive integer, and t≥2 is even, such that $\beta =t^{2}+1$. If $C_{1}$ and $C_{2}$ are two $q^{2}$-ary constacyclic codes whose defining sets are given by $Z_{1}=\cup_{i=0}^{\delta_{1}}C_{s-(q+1)i}$ and $Z_{2}=\cup_{i=0}^{\delta_{2}}C_{s-(q+1)i}$, respectively, where $0\leq \delta_{1}\leq \frac{q-\beta +t}{2\beta }$ and $\frac{q+\beta +t}{2\beta }\leq \delta_{2}\leq \frac{q^{2}+1}{2\beta }-q\delta_{1}-1$, then $C_{1}^{{⊥}_{h}}\subseteq C_{2}$.*

**Theorem 3.**  *Let $n=\frac{q^{2}+1}{\beta }$ and $s=\frac{q^{2}+1}{2}$, where q=2βm+β−t is an odd prime power with a positive integer m, and t≥2 is even, such that $\beta =t^{2}+1$. For $0\leq \delta_{1}\leq \frac{q-\beta +t}{2\beta }$ and $\frac{q+\beta +t}{2\beta }\leq \delta_{2}\leq \frac{q^{2}+1}{2\beta }-q\delta_{1}+vq-1$, then there exist asymmetric EAQMDS codes with parameters ${\left[\left[n,n-2\left(\delta_{1}+\delta_{2}+1-v\right),2\delta_{2}+2/2\delta_{1}+2;2v\right]\right]}_{q^{2}}$, where $0\leq v\leq \delta_{1}$.*

**Example 2.**  *Let t=2 and q=13, then n=34, $0\leq \delta_{1}\leq 1$, $2\leq \delta_{2}\leq 17-13\delta_{1}+13v-1$ and $0\leq v\leq \delta_{1}$. Some asymmetric EAQMDS codes derived from Theorem 3 are listed in Table 2.*

**Lemma 6.**  *Let $n=\frac{2(q^{2}+1)}{\alpha }$, $s=\frac{q^{2}+1}{2}$ and r=q+1, where q=αm+α+t or q=αm+α−t is an odd prime power and m is a positive integer, t≥3 is odd, such that $\alpha =t^{2}+1$. Then $C_{s}=\left\{s\right\}$, $C_{s+\frac{q+1}{2}n}=\left\{s+\frac{q+1}{2}n\right\}$ and $C_{s-(q+1)i}=\left\{s-\left(q+1\right)i,s+\left(q+1\right)i\right\}$ for $1\leq i\leq \frac{n}{2}-1$.*

**Proof.**  Since $s=1+\left(q+1\right)\frac{q-1}{2}$ and $s+\frac{q+1}{2}n=1+\left(q+1\right)\left(\frac{q-1}{2}+\frac{n}{2}\right)$, which implies *s*,$s+\frac{q+1}{2}n\in O_{rn}$. Since $sq^{2}=s\left(q^{2}+1-1\right)\equiv s\;\mathrm{mod}\;\left(q+1\right)n$ and $\left(s+\frac{q+1}{2}n\right)q^{2}=sq^{2}+\left(\frac{q+1}{2}n\right)\left(q^{2}+1-1\right)\equiv s+\frac{q+1}{2}n\;\mathrm{mod}\;\left(q+1\right)n$, it follows that $C_{s}=\left\{s\right\}$ and $C_{s+\frac{q+1}{2}n}=\left\{s+\frac{q+1}{2}n\right\}$. For $1\leq i\leq \frac{n}{2}-1$, it has $C_{s-(q+1)i}=\left\{s-\left(q+1\right)i,s+\left(q+1\right)i\right\}$ from $\left(s-\left(q+1\right)i\right)q^{2}=sq^{2}-\left(q+1\right)iq^{2}\equiv s+\left(q+1\right)i-\left(q+1\right)i\left(q^{2}+1\right)\equiv s+\left(q+1\right)i\;\mathrm{mod}\left(q+1\right)n$ and $\left(s+\left(q+1\right)i\right)q^{2}=sq^{2}+\left(q+1\right)iq^{2}\equiv s-\left(q+1\right)i+\left(q+1\right)i\left(q^{2}+1\right)\equiv s-\left(q+1\right)i\;\mathrm{mod}\left(q+1\right)n.$

Moreover, we show that $C_{s-(q+1)i}=\left\{s-\left(q+1\right)i,s+\left(q+1\right)i\right\}$ is disjoint for $1\leq i\leq \frac{n}{2}-1$. In fact, we assume that two integers exist, *i* and *j*, $1\leq i\neq j\leq \frac{n}{2}-1$, such that $C_{s-(q+1)i}=C_{s-(q+1)j},$ and then we have $s-\left(q+1\right)i\equiv \left(s-\left(q+1\right)j\right)q^{2k}\;\mathrm{mod}\;\left(q+1\right)n$ for k∈{0,1}.

If k=0, we have s−(q+1)i≡s−(q+1)jmod(q+1)n, which is equivalent to i=j. It is in contradiction with $1\leq i\neq j\leq \frac{n}{2}-1$.

If k=1, we have s−(q+1)i≡s+(q+1)jmod(q+1)n, which is equivalent to i+j≡nmodn. It is in contradiction with 0≤i+j≤n−2. Therefore, the result follows. □

**Lemma 7.**  *Let $n=\frac{2(q^{2}+1)}{\alpha }$, $s=\frac{q^{2}+1}{2}$ and r=q+1, where q=αm+α+t is an odd prime power with a positive integer m, and t≥3 is odd, such that $\alpha =t^{2}+1$. If $C_{1}$ and $C_{2}$ are two $q^{2}$-ary constacyclic codes whose defining sets are given by $Z_{1}=\cup_{i=0}^{\delta_{1}}C_{s-(q+1)i}$ and $Z_{2}=\cup_{i=0}^{\delta_{2}}C_{s-(q+1)i}$, respectively, where $0\leq \delta_{1}\leq \frac{q-t}{\alpha }$ and $\frac{q+\alpha -t}{\alpha }\leq \delta_{2}\leq \frac{q^{2}+1}{\alpha }-q\delta_{1}-1$, then $C_{1}^{{⊥}_{h}}\subseteq C_{2}$.*

**Proof.**  If $C_{1}$ and $C_{2}$ are two $q^{2}$-ary constacyclic codes whose defining sets are given by $Z_{1}=\cup_{i=0}^{\delta_{1}}C_{s-(q+1)i}$ and $Z_{2}=\cup_{i=0}^{\delta_{2}}C_{s-(q+1)i}$ from Lemma 6, respectively, where $0\leq \delta_{1}\leq \frac{q-t}{\alpha }$ and $\frac{q+\alpha -t}{\alpha }\leq \delta_{2}\leq \frac{q^{2}+1}{\alpha }-q\delta_{1}-1$, then $C_{1}$ and $C_{2}$ are two MDS constacyclic codes with parameters ${\left[n,n-2\delta_{1}-1,2\delta_{1}+2\right]}_{q^{2}}$ and ${\left[n,n-2\delta_{2}-1,2\delta_{2}+2\right]}_{q^{2}}$, respectively, from Propositions 1 and 2. For $0\leq \delta_{1}\leq \frac{q-t}{\alpha }$ and $\frac{q+\alpha -t}{\alpha }\leq \delta_{2}\leq \frac{q^{2}+1}{\alpha }-q\delta_{1}-1$, it has $C_{1}^{{⊥}_{h}}\subseteq C_{2}$. In fact, it only needs to show that $Z_{2}\cap -qZ_{1}=\emptyset$ for $0\leq \delta_{1}\leq \frac{q-t}{\alpha }$ and $\frac{q+\alpha -t}{\alpha }\leq \delta_{2}\leq \frac{q^{2}+1}{\alpha }-q\delta_{1}-1$. If $Z_{2}\cap -qZ_{1}\neq \emptyset$, then there exist two integers $\delta_{1}^{\prime }$ and $\delta_{2}^{\prime }$ such that $s-r\delta_{2}^{\prime }\equiv -q\left(s-r\delta_{1}^{\prime }\right)q^{2k}\;mod\;rn$ for k∈{0,1}, where $0\leq \delta_{1}^{\prime }\leq \frac{q-t}{\alpha }$ and $\frac{q+\alpha -t}{\alpha }\leq \delta_{2}^{\prime }\leq \frac{q^{2}+1}{\alpha }-q\delta_{1}^{\prime }-1$.

If k=0, it has $s-r\delta_{2}^{\prime }\equiv -q\left(s-r\delta_{1}^{\prime }\right)\equiv -qs+qr\delta_{1}^{\prime }\;mod\;rn$, i.e., $s\equiv \delta_{2}^{\prime }+q\delta_{1}^{\prime }\;mod\;n$. Since $\frac{q+\alpha -t}{\alpha }\leq \delta_{2}^{\prime }+q\delta_{1}^{\prime }\leq \frac{q^{2}+1}{\alpha }-1$, which is in contradiction with $s\equiv \delta_{2}^{\prime }+q\delta_{1}^{\prime }\;mod\;n$.

If k=1, it has $s-r\delta_{2}^{\prime }\equiv -\left(s-r\delta_{1}^{\prime }\right)q^{3}\equiv -qs+rq^{3}\delta_{1}^{\prime }\;mod\;rn$, i.e., $s\equiv \delta_{2}^{\prime }-q\delta_{1}^{\prime }\;mod\;n$. If $\delta_{1}^{\prime }=0$, then it has $\delta_{2}^{\prime }\equiv s\;mod\;n$ that is in contradiction with $\frac{q+\alpha -t}{\alpha }\leq \delta_{2}^{\prime }\leq \frac{q^{2}+1}{\alpha }-q\delta_{1}^{\prime }-1$. Now we consider that $s+q\delta_{1}^{\prime }\equiv \delta_{2}^{\prime }\;mod\;n$ for $1\leq \delta_{1}^{\prime }\leq \frac{q-t}{\alpha }$ and $\frac{q+\alpha -t}{\alpha }\leq \delta_{2}^{\prime }\leq \frac{q^{2}+1}{\alpha }-q\delta_{1}^{\prime }-1$. Since $s+q\delta_{1}^{\prime }\equiv \delta_{2}^{\prime }\;mod\;n$ is equivalent to $\frac{q^{2}+1}{\alpha }+q\delta_{1}^{\prime }\equiv \delta_{2}^{\prime }\;mod\;n$; moreover, it has $\frac{q^{2}+1}{\alpha }+q\leq \frac{q^{2}+1}{\alpha }+q\delta_{1}^{\prime }\leq \frac{q^{2}+1}{\alpha }+q\left(\frac{q-t}{\alpha }\right)=\frac{q^{2}-qt+1}{\alpha }$, which is in contradiction with $\frac{q+\alpha -t}{\alpha }\leq \delta_{2}^{\prime }\leq \frac{q^{2}+1}{\alpha }-q\delta_{1}^{\prime }-1\leq \frac{q^{2}+1}{\alpha }-q-1$. Therefore, it has $C_{1}^{{⊥}_{h}}\subseteq C_{2}$. □

**Theorem 4.**  *Let $n=\frac{2(q^{2}+1)}{\alpha }$ and $s=\frac{q^{2}+1}{2}$, where q=αm+α+t is an odd prime power with a positive integer m, t≥3 is odd, such that $\alpha =t^{2}+1$. For $0\leq \delta_{1}\leq \frac{q-t}{\alpha }$ and $\frac{q+\alpha -t}{\alpha }\leq \delta_{2}\leq \frac{q^{2}+1}{\alpha }-q\delta_{1}+vq-1$, then there exist asymmetric EAQMDS codes with parameters ${\left[\left[n,n-2\left(\delta_{1}+\delta_{2}+1-v\right),2\delta_{2}+2/2\delta_{1}+2;2v\right]\right]}_{q^{2}}$, where $0\leq v\leq \frac{t-1}{2}$.*

**Proof.**  If $C_{1}$ and $C_{2}$ are two $q^{2}$-ary constacyclic codes whose defining sets are given by $Z_{1}=\cup_{i=0}^{\delta_{1}}C_{s-(q+1)i}$ and $Z_{2}=\cup_{i=0}^{\delta_{2}}C_{s-(q+1)i}$, respectively, where $0\leq \delta_{1}\leq \frac{q-t}{\alpha }$ and $\frac{q+\alpha -t}{\alpha }\leq \delta_{2}\leq \frac{q^{2}+1}{\alpha }-q\delta_{1}+vq-1$, then $C_{1}$ and $C_{2}$ are two MDS constacyclic codes with parameters ${\left[n,n-2\delta_{1}-1,2\delta_{1}+2\right]}_{q^{2}}$ and ${\left[n,n-2\delta_{2}-1,2\delta_{2}+2\right]}_{q^{2}}$, respectively, from Propositions 1 and 2. From Lemma 7, we can see that $C_{1}^{{⊥}_{h}}\subseteq C_{2}$ for $0\leq \delta_{1}\leq \frac{q-t}{\alpha }$ and $\frac{q+\alpha -t}{\alpha }\leq \delta_{2}\leq \frac{q^{2}+1}{\alpha }-q\delta_{1}-1$, which implies that v=0. At this point, the asymmetric EAQMDS codes degenerates into asymmetric quantum MDS codes.

Now, we consider the case v≥1, and the range $\delta_{2}$ can be divided into some intervals with $I_{0}=\left[\frac{q+\alpha -t}{\alpha },\frac{q^{2}+1}{\alpha }-q\delta_{1}-1\right]$, $I_{1}=\left[\frac{q^{2}+1}{\alpha }-q\delta_{1},\frac{q^{2}+1}{\alpha }-q\delta_{1}+q-1\right],$ $I_{2}=\left[\frac{q^{2}+1}{\alpha }-q\delta_{1}+q,\frac{q^{2}+1}{\alpha }-q\delta_{1}+2q-1\right],$…, $I_{v}=\left[\frac{q^{2}+1}{\alpha }-q\delta_{1}+\left(v-1\right)q,\frac{q^{2}+1}{\alpha }-q\delta_{1}+vq-1\right],$ where $1\leq v\leq \frac{t-1}{2}$.

In order to obtain the number of entangled states, it only needs to show that $|Z_{11}\left|\;=\;\right|Z_{1}\cap \left(-qZ_{2}\right)|\;=2v$ (or $|Z_{21}\left|\;=\;\right|Z_{2}\cap \left(-qZ_{1}\right)|\;=2v$) from Lemma 2. Hence,

(38) $$\begin{array}{cc} \; & -qZ_{1}\cap Z_{2}\\ \; & =-q\left(\cup_{i=0}^{\delta_{1}}C_{s-(q+1)i}\right)\cap \left(\cup_{j=0}^{\delta_{2}}C_{s-(q+1)j}\right)\\ \; & =-q\left(\cup_{i=0}^{\delta_{1}}C_{s-(q+1)i}\right)\cap \left(\cup_{l=0}^{v}\cup_{j\in I_{l}}C_{s-(q+1)j}\right)\\ \; & =\left(-q\cup_{i=0}^{\delta_{1}}C_{s-(q+1)i}\cap \left(\cup_{j\in I_{0}}C_{s-(q+1)j}\right)\right)\cup \left(-q\cup_{i=0}^{\delta_{1}}C_{s-(q+1)i}\cap \left(\cup_{j\in I_{1}}C_{s-(q+1)j}\right)\right)\cup \\ \; & \ldots \cup (-q\cup_{i=0}^{\delta_{1}}C_{s-(q+1)i}\cap \left(\cup_{j\in I_{v}}C_{s-(q+1)j}\right)) \end{array}$$

Here, we only discuss the case $I_{v}$(v≥1) as follows; other cases can be discussed in a similar manner.

If $I_{v}=\left[\frac{q^{2}+1}{\alpha }-q\delta_{1}+\left(v-1\right)q,\frac{q^{2}+1}{\alpha }-q\delta_{1}+vq-1\right],$ then

(39) $$\begin{array}{cc} \; & -q(s-\left(q+1\right)\left(\frac{q^{2}+1}{\alpha }-q\delta_{1}+\left(v-1\right)q\right))\\ & \equiv -\left(q+1\right)s+s+q\left(q+1\right)\frac{q^{2}+1}{\alpha }-q^{2}\left(q+1\right)\left(\delta_{1}-v+1\right)\\ \; & \equiv s-\left(q+1\right)s+\left(q^{2}-1+q+1\right)\frac{q^{2}+1}{\alpha }-\left(q^{2}+1-1\right)\left(q+1\right)\left(\delta_{1}-v+1\right)\\ \; & \equiv s-\left(q+1\right)s+\left(q+1\right)\frac{q^{2}+1}{\alpha }+\left(q+1\right)\left(\delta_{1}-v+1\right)\\ \; & \equiv s-\left(q+1\right)\left(\frac{q^{2}+1}{2}-\frac{q^{2}+1}{\alpha }-\delta_{1}+v-1\right)\\ \; & \equiv s-\left(q+1\right)\left(\frac{\left(q^{2}+1\right)\left(\alpha -2\right)}{2\alpha }-\delta_{1}+v-1\right)\\ \; & \equiv s+\left(q+1\right)\left(\delta_{1}-v+1\right)mod\left(q+1\right)n, \end{array}$$

and

(40) $$\begin{array}{cc} \; & -q(s+\left(q+1\right)\left(\frac{q^{2}+1}{\alpha }-q\delta_{1}+q\left(v-1\right)\right))\\ \; & \equiv -\left(q+1\right)s+s-q\left(q+1\right)\frac{q^{2}+1}{\alpha }+q^{2}\left(q+1\right)\left(\delta_{1}-v+1\right)\\ \; & \equiv s-\left(q+1\right)s-\left(q+1\right)\frac{q^{2}+1}{\alpha }-\left(q+1\right)\left(\delta_{1}-v+1\right)\\ \; & \equiv s-\left(q+1\right)\left(\frac{q^{2}+1}{2}+\frac{q^{2}+1}{\alpha }+\delta_{1}-v+1\right)\\ \; & \equiv s-\left(q+1\right)\left(\frac{\left(q^{2}+1\right)\left(\alpha +2\right)}{2\alpha }+\delta_{1}-v+1\right)\\ \; & \equiv s-\left(q+1\right)\left(\delta_{1}-v+1\right)mod\left(q+1\right)n, \end{array}$$

which implies $-qC_{s-\left(q+1\right)(\frac{q^{2}+1}{\alpha }-q\delta_{1}+q\left(v-1\right))}=C_{s-\left(q+1\right)\left(\delta_{1}-v+1\right)}$.

Moreover,

(41) $$\begin{array}{cc} \; & -q\cup_{i=0}^{\delta_{1}}C_{s-(q+1)i}\cap \left(\cup_{j\in I_{v}}C_{s-(q+1)j}\right)\\ \; & =-q\left(\cup_{i=0}^{\delta_{1}}C_{s-(q+1)i}\right)\cap \left(\cup_{j=\frac{q^{2}+1}{\alpha }-q\delta_{1}+\left(v-1\right)q}^{\frac{q^{2}+1}{\alpha }-q\delta_{1}+vq-1}C_{s-(q+1)j}\right)\\ \; & =-q\left(\cup_{i=0}^{\delta_{1}}C_{s-(q+1)i}\right)\cap \left(\cup_{j=\frac{q^{2}+1}{\alpha }-q\delta_{1}+\left(v-1\right)q+1}^{\frac{q^{2}+1}{\alpha }-q\delta_{1}+vq-1}C_{s-(q+1)j}\cup C_{s-\left(q+1\right)(\frac{q^{2}+1}{\alpha }-q\delta_{1}+\left(v-1\right)q)}\right)\\ \; & =(-q\left(\cup_{i=0}^{\delta_{1}}C_{s-(q+1)i}\right)\cap \left(\cup_{j=\frac{q^{2}+1}{\alpha }-q\delta_{1}+\left(v-1\right)q+1}^{\frac{q^{2}+1}{\alpha }-q\delta_{1}+vq-1}C_{s-(q+1)j}\right))\\ \; & \cup (-q\left(\cup_{i=0}^{\delta_{1}}C_{s-(q+1)i}\right)\cap C_{s-\left(q+1\right)(\frac{q^{2}+1}{\alpha }-q\delta_{1}+q\left(v-1\right))}) \end{array}$$

From Lemma 7, we can see that

(42) $$-q\left(\cup_{i=0}^{\delta_{1}}C_{s-(q+1)i}\right)\cap C_{s-\left(q+1\right)(\frac{q^{2}+1}{\alpha }-q\delta_{1}+q\left(v-1\right))})=C_{s-\left(q+1\right)\left(\delta_{1}-v+1\right)}.$$

Hence, Formula (41) is equivalent to

(43) $$\begin{array}{cc} \; & -q\cup_{i=0}^{\delta_{1}}C_{s-(q+1)i}\cap \left(\cup_{j\in I_{v}}C_{s-(q+1)j}\right)\\ \; & =-q\left(\cup_{i=0}^{\delta_{1}}C_{s-(q+1)i}\right)\cap \left(\cup_{j=\frac{q^{2}+1}{\alpha }-q\delta_{1}+\left(v-1\right)q+1}^{\frac{q^{2}+1}{\alpha }-q\delta_{1}+vq-1}\right)\cup C_{s-\left(q+1\right)\left(\delta_{1}-v+1\right)}. \end{array}$$

Next, we will prove that if $-q\left(\cup_{i=0}^{\delta_{1}}C_{s-(q+1)i}\right)\cap \left(\cup_{j=\frac{q^{2}+1}{\alpha }-q\delta_{1}+\left(v-1\right)q+1}^{\frac{q^{2}+1}{\alpha }-q\delta_{1}+vq-1}\right)=\emptyset$ holds, then the equation

(44) $$\begin{array}{c} -q\cup_{i=0}^{\delta_{1}}C_{s-(q+1)i}\cap \left(\cup_{j\in I_{0}}C_{s-(q+1)j}\right)=C_{s-\left(q+1\right)\left(\delta_{1}-v+1\right)} \end{array}$$

is true.

Assume that $-q\left(\cup_{i=0}^{\delta_{1}}C_{s-(q+1)i}\right)\cap \left(\cup_{j=\frac{q^{2}+1}{\alpha }-q\delta_{1}+\left(v-1\right)q+1}^{\frac{q^{2}+1}{\alpha }-q\delta_{1}+vq-1}C_{s-(q+1)j}\right)\neq \emptyset$, then there exist two integers $\delta_{1}^{\prime }$ and $\delta_{2}^{\prime }$ such that $s-\left(q+1\right)\delta_{2}^{\prime }\equiv -q\left(s-\left(q+1\right)\delta_{1}^{\prime }\right)q^{2k}\;mod\;\left(q+1\right)n$ for k∈{0,1}, where $0\leq \delta_{1}^{\prime }\leq \frac{q-t}{\alpha }$ and $\frac{q^{2}+1}{\alpha }-q\delta_{1}^{\prime }+\left(v-1\right)q+1\leq \delta_{2}^{\prime }\leq \frac{q^{2}+1}{\alpha }-q\delta_{1}^{\prime }+vq-1$.

If k=0, it has $s-\left(q+1\right)\delta_{2}^{\prime }\equiv -q\left(s-\left(q+1\right)\delta_{1}^{\prime }\right)\equiv -qs+qr\delta_{1}^{\prime }\;mod\;\left(q+1\right)n$, i.e., $s\equiv \delta_{2}^{\prime }+q\delta_{1}^{\prime }\;mod\;n$. Since $\frac{q^{2}+1}{\alpha }+\left(v-1\right)q+1\leq \delta_{2}^{\prime }+q\delta_{1}^{\prime }\leq \frac{q^{2}+1}{\alpha }+vq-1$, which is in contradiction with $s\equiv \delta_{2}^{\prime }+q\delta_{1}^{\prime }\;mod\;n$.

If k=1, it has $s-\left(q+1\right)\delta_{2}^{\prime }\equiv -\left(s-\left(q+1\right)\delta_{1}^{\prime }\right)q^{3}\equiv -qs+rq^{3}\delta_{1}^{\prime }\;mod\;\left(q+1\right)n$, i.e., $s\equiv \delta_{2}^{\prime }-q\delta_{1}^{\prime }\;mod\;n$. If $\delta_{1}^{\prime }=0$, then it has $\delta_{2}^{\prime }\equiv s\;mod\;n$ that is in contradiction with $\frac{q^{2}+1}{\alpha }+\left(v-1\right)q+1\leq \delta_{2}^{\prime }\leq \frac{q^{2}+1}{\alpha }+vq-1$. Now we consider that $s+q\delta_{1}^{\prime }\equiv \delta_{2}^{\prime }\;mod\;n$ for $1\leq \delta_{1}^{\prime }\leq \frac{q-t}{\alpha }$ and $\frac{q^{2}+1}{\alpha }-q\delta_{1}^{\prime }+\left(v-1\right)q+1\leq \delta_{2}^{\prime }\leq \frac{q^{2}+1}{\alpha }-q\delta_{1}^{\prime }+vq-1$. Since $s+q\delta_{1}^{\prime }\equiv \delta_{2}^{\prime }\;mod\;n$ is equivalent to $\frac{q^{2}+1}{\alpha }+q\delta_{1}^{\prime }\equiv \delta_{2}^{\prime }\;mod\;n$, moreover, it has $\frac{q^{2}+1}{\alpha }+q\leq \frac{q^{2}+1}{\alpha }+q\delta_{1}^{\prime }\leq \frac{q^{2}+1}{\alpha }+q\left(\frac{q-t}{\alpha }\right)=\frac{2q^{2}-tq+1}{\alpha }$, which is in contradiction with $\frac{q^{2}+1}{\alpha }-q\delta_{1}^{\prime }+\left(v-1\right)q-1\leq \delta_{2}^{\prime }\leq \frac{q^{2}+1}{\alpha }-q\left(\delta_{1}^{\prime }-v\right)-1$. Therefore, it has $-q\left(\cup_{i=0}^{\delta_{1}}C_{s-(q+1)i}\right)\cap \left(\cup_{i=\frac{q^{2}+1}{\alpha }-q\delta_{1}+\left(v-1\right)q+1}^{\frac{q^{2}+1}{\alpha }-q\delta_{1}+vq-1}C_{s-(q+1)i}\right)=\emptyset .$

According to the above discussion, the number of entangled states is 2v, where $0\leq v\leq \frac{t-1}{2}$. Therefore, we can see that asymmetric EAQMDS codes with parameters ${\left[\left[n,n-2\left(\delta_{1}+\delta_{2}+1-v\right),2\delta_{2}+2/2\delta_{1}+2;2v\right]\right]}_{q^{2}}$ exist from Theorem 1 and Proposition 3. □

**Example 3.**  *Let t=3 and q=23, then n=106, $0\leq \delta_{1}\leq 2$, $3\leq \delta_{2}\leq 53-23\delta_{1}+23v-1$ and 0≤v≤1. Some asymmetric EAQMDS codes derived from Theorem 4 are listed in Table 3.*

Similar to Lemma 7 and Theorem 4, we can also obtain the following Lemma 8 and Theorem 5.

**Lemma 8.**  *Let $n=\frac{2(q^{2}+1)}{\alpha }$ and $s=\frac{q^{2}+1}{2}$, where q=αm+α−t is an odd prime power with a positive integer m, and t≥3 is odd, such that $\alpha =t^{2}+1$. If $C_{1}$ and $C_{2}$ are two $q^{2}$-ary constacyclic codes whose defining sets are given by $Z_{1}=\cup_{i=0}^{\delta_{1}}C_{s-(q+1)i}$ and $Z_{2}=\cup_{i=0}^{\delta_{2}}C_{s-(q+1)i}$, respectively, where $0\leq \delta_{1}\leq \frac{q+t}{\alpha }$ and $\frac{q+\alpha +t}{\alpha }\leq \delta_{2}\leq \frac{q^{2}+1}{\alpha }-q\delta_{1}-1$, then $C_{1}^{{⊥}_{h}}\subseteq C_{2}$.*

**Theorem 5.**  *Let $n=\frac{2(q^{2}+1)}{\alpha }$ and $s=\frac{q^{2}+1}{2}$, where q=αm+α−t is an odd prime power with a positive integer m, and t≥3 is odd, such that $\alpha =t^{2}+1$. For $0\leq \delta_{1}\leq \frac{q+t}{\alpha }$ and $\frac{q+\alpha +t}{\alpha }\leq \delta_{2}\leq \frac{q^{2}+1}{\alpha }-q\delta_{1}+vq-1$, then there exist asymmetric EAQMDS codes with parameters ${\left[\left[n,n-2\left(\delta_{1}+\delta_{2}+1-v\right),2\delta_{2}+2/2\delta_{1}+2;2v\right]\right]}_{q^{2}}$, where $0\leq v\leq \frac{t-1}{2}$.*

**Example 4.**  *Let t=3 and q=27, then n=146, $0\leq \delta_{1}\leq 3$, $4\leq \delta_{2}\leq 73-27\delta_{1}+27v-1$ and 0≤v≤1. Some asymmetric EAQMDS codes derived from Theorem 5 are listed in Table 4.*

## 5. Codes Comparison

In this paper, the constacyclic codes with lengths $\frac{q^{2}+1}{\beta }$ and $\frac{2(q^{2}+1)}{\alpha }$ are utilized to construct some classes of asymmetric EAQMDS codes. Current studies [75,76,77] indicate that the energy relaxation time is much larger than the phase coherence time, indicating that phase-shift errors occur more frequently than qubit-flip (or qudit-flip) errors. From Table 5, Table 6, Table 7 and Table 8, the distances of some codes constructed in this paper are far beyond q+1, which indicates that asymmetric EAQMDS codes constructed in this paper have greater asymmetry and stronger capabilities for detecting and correcting phase-shift errors.

In addition, we compare the asymmetric EAQMDS codes constructed in this paper with respect to the ones constructed in [18], because the codes constructed in that paper have better parameters than the symmetric EAQMDS codes in the other current references [25,27,28]. In Table 5, Table 6, Table 7 and Table 8, we compare the asymmetric EAQMDS codes constructed in this paper with respect to the ones in [18]. Although some asymmetric EAQMDS codes constructed in this paper have the same net rate (k−c)/n as those constructed in [18], it can be found that the ones in [18] require the use of a larger number of entangled states, which also implies that preparing entangled states for asymmetric EAQMDS codes in [18] require more effort and cost. Furthermore, it can be found from Table 5, Table 6, Table 7 and Table 8 that under the same conditions of code length, $d_{z}$ and $d_{x}$, this paper can construct some asymmetric EAQMDS codes with higher net rates that are not achieved in [18]. Finally, the parameters of the asymmetric EAQMDS codes constructed in this paper are more general than the quantum asymmetric codes constructed in [20,21,22,23,24,26], and some quantum codes have better parameters than the quantum asymmetric codes constructed using generalized RS codes in [29]. For example, the quantum codes ${\left[\left[96;1,93/4\right]\right]}_{23^{2}}$ and ${\left[\left[140;2,136/4\right]\right]}_{27^{2}}$ constructed in [29] are compared to the ones ${\left[\left[106;12,94/4\right]\right]}_{23^{2}}$ and $[{\left[146,10,136/4;2\right]}_{27^{2}}$ constructed in this paper. The code rate of the quantum codes constructed in this paper is higher than the code rate of the quantum codes constructed in [29]. Here, we only consider the case where c=2. The same situation applies to other quantum codes with the parameters where c=2v and v≥2.

## 6. Conclusions and Discussions

In this paper, we construct four families of asymmetric EAQMDS codes from constacyclic codes. We find that pre-sharing a certain number of entangled states between communicating parties allows asymmetric QEC codes to be no longer constrained by the Hermitian dual-containing condition. As a result, this can enhance the error correction capabilities of asymmetric EAQMDS codes from Theorems 2–5. Although the finite fields used to construct asymmetric EAQMDS codes in this paper are larger than the ones used in [18], this paper obtains some asymmetric EAQMDS codes with a small number of entangled states that cannot be constructed in [18]. How to construct asymmetric EAQMDS codes with good performance on small finite fields is the next important issue that we need to explore. Although the Hull of generalized RS codes has been used to construct EAQMDS codes [47], the method of the decomposition of the defining set is more intuitive and clearer. If one wants to obtain more asymmetric EAQMDS codes with other entanglements, one can use the same method in this paper to obtain them. Finally, exploring how to use combinatorial methods to obtain asymmetric EAQMDS codes with flexible entangled states presents an intriguing area for future work.

## Figures and Tables

**Table 1:** Sample parameters of asymmetric EAQMDS codes constructed from Theorem 2.

*q*	*n*	${\left[\left[n,k,d_{z}/d_{x};c\right]\right]}_{q^{2}}$
17	58	${\left[\left[58,2,56/4;2\right]\right]}_{17^{2}}$
17	58	${\left[\left[58,4,54/4;2\right]\right]}_{17^{2}}$
17	58	${\left[\left[58,6,52/4;2\right]\right]}_{17^{2}}$
17	58	${\left[\left[58,8,50/4;2\right]\right]}_{17^{2}}$
17	58	${\left[\left[58,10,48/4;2\right]\right]}_{17^{2}}$
17	58	${\left[\left[58,12,46/4;2\right]\right]}_{17^{2}}$
17	58	${\left[\left[58,14,44/4;2\right]\right]}_{17^{2}}$
17	58	${\left[\left[58,16,42/4;2\right]\right]}_{17^{2}}$
17	58	${\left[\left[58,18,40/4;2\right]\right]}_{17^{2}}$
…	…	…
17	58	${\left[\left[58,40,14/4;2\right]\right]}_{17^{2}}$
17	58	${\left[\left[58,42,12/4;2\right]\right]}_{17^{2}}$
17	58	${\left[\left[58,44,10/4;2\right]\right]}_{17^{2}}$
17	58	${\left[\left[58,48,8/4;2\right]\right]}_{17^{2}}$
17	58	${\left[\left[58,52,6/4;2\right]\right]}_{17^{2}}$

**Table 2:** Sample parameters of asymmetric EAQMDS codes constructed from Theorem 3.

*q*	*n*	${\left[\left[n,k,d;c\right]\right]}_{q^{2}}$
13	34	${\left[\left[34,2,32/4;2\right]\right]}_{13^{2}}$
13	34	${\left[\left[34,4,30/4;2\right]\right]}_{13^{2}}$
13	34	${\left[\left[34,6,28/4;2\right]\right]}_{13^{2}}$
13	34	${\left[\left[34,8,26/4;2\right]\right]}_{13^{2}}$
13	34	${\left[\left[34,10,24/4;2\right]\right]}_{13^{2}}$
13	34	${\left[\left[34,12,22/4;2\right]\right]}_{13^{2}}$
…	…	…
13	34	${\left[\left[34,20,14/4;2\right]\right]}_{13^{2}}$
13	34	${\left[\left[34,22,12/4;2\right]\right]}_{13^{2}}$
13	34	${\left[\left[34,24,10/4;2\right]\right]}_{13^{2}}$
13	34	${\left[\left[34,26,8/4;2\right]\right]}_{13^{2}}$
13	34	${\left[\left[34,28,6/4;2\right]\right]}_{13^{2}}$

**Table 3:** Sample parameters of asymmetric EAQMDS codes constructed from Theorem 4.

*q*	*n*	${\left[\left[n,k,d_{z}/d_{x};c\right]\right]}_{q^{2}}$
23	106	${\left[\left[106,98,8/4;2\right]\right]}_{23^{2}}$
23	106	${\left[\left[106,96,10/4;2\right]\right]}_{23^{2}}$
23	106	${\left[\left[106,94,12/4;2\right]\right]}_{23^{2}}$
23	106	${\left[\left[106,92,14/4;2\right]\right]}_{23^{2}}$
…	…	…
23	106	${\left[\left[106,14,92/4;2\right]\right]}_{23^{2}}$
23	106	${\left[\left[106,12,94/4;2\right]\right]}_{23^{2}}$
23	106	${\left[\left[106,10,96/4;2\right]\right]}_{23^{2}}$
23	106	${\left[\left[106,8,98/4;2\right]\right]}_{23^{2}}$
23	106	${\left[\left[106,6,100/4;2\right]\right]}_{23^{2}}$
23	106	${\left[\left[106,4,102/4;2\right]\right]}_{23^{2}}$
23	106	${\left[\left[106,2,104/4;2\right]\right]}_{23^{2}}$

**Table 4:** Sample parameters of asymmetric EAQMDS codes constructed from Theorem 5.

*q*	*n*	${\left[\left[n,k,d_{z}/d_{x};c\right]\right]}_{q^{2}}$
27	146	${\left[\left[146,136,10/4;2\right]\right]}_{27^{2}}$
27	146	${\left[\left[146,134,12/4;2\right]\right]}_{27^{2}}$
27	146	${\left[\left[146,132,14/4;2\right]\right]}_{27^{2}}$
27	146	${\left[\left[146,130,16/4;2\right]\right]}_{27^{2}}$
…	…	…
27	146	${\left[\left[146,12,134/4;2\right]\right]}_{27^{2}}$
27	146	${\left[\left[146,10,136/4;2\right]\right]}_{27^{2}}$
27	146	${\left[\left[146,8,138/4;2\right]\right]}_{27^{2}}$
27	146	${\left[\left[146,6,140/4;2\right]\right]}_{27^{2}}$
27	146	${\left[\left[146,4,142/4;2\right]\right]}_{27^{2}}$
27	146	${\left[\left[146,2,144/4;2\right]\right]}_{27^{2}}$

**Table 5:** Comparison of asymmetric EAQMDS codes derived from Theorem 2 with those found in [18].

Codes in [18]	(k−c)/n	Codes from Theorem 2	(k−c)/n
${\left[\left[58,6,54/4;4\right]\right]}_{17^{2}}$	0.035	${\left[\left[58,4,54/4;2\right]\right]}_{17^{2}}$	0.035
${\left[\left[58,12,52/4;8\right]\right]}_{17^{2}}$	0.069	${\left[\left[58,6,52/4;2\right]\right]}_{17^{2}}$	0.069
${\left[\left[58,18,50/4;12\right]\right]}_{17^{2}}$	0.103	${\left[\left[58,8,50/4;2\right]\right]}_{17^{2}}$	0.103
${\left[\left[58,24,48/4;16\right]\right]}_{17^{2}}$	0.138	${\left[\left[58,10,48/4;2\right]\right]}_{17^{2}}$	0.138
${\left[\left[58,30,46/4;20\right]\right]}_{17^{2}}$	0.172	${\left[\left[58,12,46/4;2\right]\right]}_{17^{2}}$	0.172
${\left[\left[58,36,44/4;24\right]\right]}_{17^{2}}$	0.207	${\left[\left[58,14,44/4;2\right]\right]}_{17^{2}}$	0.207
${\left[\left[58,42,42/4;28\right]\right]}_{17^{2}}$	0.241	${\left[\left[58,16,42/4;2\right]\right]}_{17^{2}}$	0.241
${\left[\left[58,48,40/4;32\right]\right]}_{17^{2}}$	0.276	${\left[\left[58,18,40/4;2\right]\right]}_{17^{2}}$	0.276
${\left[\left[58,54,38/4;36\right]\right]}_{17^{2}}$	0.310	${\left[\left[58,20,38/4;2\right]\right]}_{17^{2}}$	0.310
—	—	${\left[\left[58,22,36/4;2\right]\right]}_{17^{2}}$	0.345
—	—	${\left[\left[58,24,34/4;2\right]\right]}_{17^{2}}$	0.379
—	—	${\left[\left[58,26,32/4;2\right]\right]}_{17^{2}}$	0.414
—	—	${\left[\left[58,28,30/4;2\right]\right]}_{17^{2}}$	0.448
—	—	${\left[\left[58,30,28/4;2\right]\right]}_{17^{2}}$	0.483
—	—	${\left[\left[58,32,26/4;2\right]\right]}_{17^{2}}$	0.517
—	—	${\left[\left[58,34,24/4;2\right]\right]}_{17^{2}}$	0.552
—	—	${\left[\left[58,36,22/4;2\right]\right]}_{17^{2}}$	0.586
—	—	${\left[\left[58,38,20/4;2\right]\right]}_{17^{2}}$	0.621
—	—	${\left[\left[58,40,18/4;2\right]\right]}_{17^{2}}$	0.655
—	—	${\left[\left[58,42,16/4;2\right]\right]}_{17^{2}}$	0.690
—	—	${\left[\left[58,44,14/4;2\right]\right]}_{17^{2}}$	0.724
—	—	${\left[\left[58,46,12/4;2\right]\right]}_{17^{2}}$	0.759
—	—	${\left[\left[58,48,10/4;2\right]\right]}_{17^{2}}$	0.793
—	—	${\left[\left[58,50,8/4;2\right]\right]}_{17^{2}}$	0.828
—	—	${\left[\left[58,52,6/4;2\right]\right]}_{17^{2}}$	0.862

**Table 6:** Comparison of asymmetric EAQMDS codes derived from Theorem 3 with those found in [18].

Codes in [18]	(k−c)/n	Codes from Theorem 3	(k−c)/n
${\left[\left[34,6,30/4;4\right]\right]}_{13^{2}}$	0.059	${\left[\left[34,4,30/4;2\right]\right]}_{13^{2}}$	0.059
${\left[\left[34,12,28/4;8\right]\right]}_{13^{2}}$	0.118	${\left[\left[34,6,28/4;2\right]\right]}_{13^{2}}$	0.118
${\left[\left[34,18,26/4;12\right]\right]}_{13^{2}}$	0.177	${\left[\left[34,8,24/4;2\right]\right]}_{13^{2}}$	0.177
${\left[\left[34,24,24/4;16\right]\right]}_{13^{2}}$	0.235	${\left[\left[34,10,24/4;2\right]\right]}_{13^{2}}$	0.235
${\left[\left[34,30,22/4;20\right]\right]}_{13^{2}}$	0.294	${\left[\left[34,12,22/4;2\right]\right]}_{13^{2}}$	0.294
—	—	${\left[\left[34,14,20/4;2\right]\right]}_{13^{2}}$	0.353
—	—	${\left[\left[34,16,18/4;2\right]\right]}_{13^{2}}$	0.412
—	—	${\left[\left[34,18,16/4;2\right]\right]}_{13^{2}}$	0.471
—	—	${\left[\left[34,20,14/4;2\right]\right]}_{13^{2}}$	0.529
—	—	${\left[\left[34,22,12/4;2\right]\right]}_{13^{2}}$	0.588
—	—	${\left[\left[34,24,10/4;2\right]\right]}_{13^{2}}$	0.647
—	—	${\left[\left[34,26,8/4;2\right]\right]}_{13^{2}}$	0.706
—	—	${\left[\left[34,28,6/4;2\right]\right]}_{13^{2}}$	0.765

**Table 7:** Comparison of asymmetric EAQMDS codes derived from Theorem 4 with those found in [18].

Codes in [18]	(k−c)/n	Codes from Theorem 4	(k−c)/n
${\left[\left[106,6,102/4;4\right]\right]}_{23^{2}}$	0.019	${\left[\left[106,4,102/4;2\right]\right]}_{23^{2}}$	0.019
${\left[\left[106,12,100/4;8\right]\right]}_{23^{2}}$	0.038	${\left[\left[106,6,100/4;2\right]\right]}_{23^{2}}$	0.038
${\left[\left[106,18,98/4;12\right]\right]}_{23^{2}}$	0.057	${\left[\left[106,8,98/4;2\right]\right]}_{23^{2}}$	0.057
${\left[\left[106,24,96/4;16\right]\right]}_{23^{2}}$	0.076	${\left[\left[106,10,96/4;2\right]\right]}_{23^{2}}$	0.076
${\left[\left[106,30,94/4;20\right]\right]}_{23^{2}}$	0.094	${\left[\left[106,12,94/4;2\right]\right]}_{23^{2}}$	0.094
${\left[\left[106,36,92/4;24\right]\right]}_{23^{2}}$	0.113	${\left[\left[106,14,92/4;2\right]\right]}_{23^{2}}$	0.113
${\left[\left[106,42,90/4;28\right]\right]}_{23^{2}}$	0.132	${\left[\left[106,16,90/4;2\right]\right]}_{23^{2}}$	0.132
${\left[\left[106,48,88/4;32\right]\right]}_{23^{2}}$	0.151	${\left[\left[106,18,88/4;2\right]\right]}_{23^{2}}$	0.151
${\left[\left[106,54,86/4;36\right]\right]}_{23^{2}}$	0.151	${\left[\left[106,18,86/4;2\right]\right]}_{23^{2}}$	0.151
…	…	…	…
${\left[\left[106,90,74/4;60\right]\right]}_{23^{2}}$	0.283	${\left[\left[106,32,74/4;2\right]\right]}_{23^{2}}$	0.283
${\left[\left[106,96,72/4;64\right]\right]}_{23^{2}}$	0.302	${\left[\left[106,34,72/4;2\right]\right]}_{23^{2}}$	0.302
${\left[\left[106,102,70/4;68\right]\right]}_{23^{2}}$	0.321	${\left[\left[106,36,70/4;2\right]\right]}_{23^{2}}$	0.321
—	—	${\left[\left[106,38,68/4;2\right]\right]}_{23^{2}}$	0.340
…	…	…	…
—	—	${\left[\left[106,88,18/4;2\right]\right]}_{23^{2}}$	0.811
—	—	${\left[\left[106,90,16/4;2\right]\right]}_{23^{2}}$	0.830
—	—	${\left[\left[106,92,14/4;2\right]\right]}_{23^{2}}$	0.849
—	—	${\left[\left[106,94,12/4;2\right]\right]}_{23^{2}}$	0.868
—	—	${\left[\left[106,96,10/4;2\right]\right]}_{23^{2}}$	0.887
—	—	${\left[\left[106,98,8/4;2\right]\right]}_{23^{2}}$	0.906

**Table 8:** Comparison of asymmetric EAQMDS codes derived from Theorem 5 with those found in [18].

Codes in [18]	(k−c)/n	Codes from Theorem 5	(k−c)/n
${\left[\left[146,6,142/4;4\right]\right]}_{27^{2}}$	0.014	${\left[\left[146,4,142/4;2\right]\right]}_{27^{2}}$	0.014
${\left[\left[146,12,140/4;8\right]\right]}_{27^{2}}$	0.027	${\left[\left[146,6,140/4;2\right]\right]}_{27^{2}}$	0.027
${\left[\left[146,18,138/4;12\right]\right]}_{27^{2}}$	0.041	${\left[\left[146,8,138/4;2\right]\right]}_{27^{2}}$	0.041
${\left[\left[146,24,136/4;16\right]\right]}_{27^{2}}$	0.055	${\left[\left[146,10,136/4;2\right]\right]}_{27^{2}}$	0.055
${\left[\left[146,30,134/4;20\right]\right]}_{27^{2}}$	0.069	${\left[\left[146,12,134/4;2\right]\right]}_{27^{2}}$	0.069
${\left[\left[146,36,132/4;24\right]\right]}_{27^{2}}$	0.082	${\left[\left[146,14,132/4;2\right]\right]}_{27^{2}}$	0.822
${\left[\left[146,42,130/4;28\right]\right]}_{27^{2}}$	0.096	${\left[\left[146,16,130/4;2\right]\right]}_{27^{2}}$	0.096
…	…	…	…
${\left[\left[146,126,102/4;84\right]\right]}_{27^{2}}$	0.288	${\left[\left[146,44,102/4;2\right]\right]}_{27^{2}}$	0.288
${\left[\left[146,132,100/4;88\right]\right]}_{27^{2}}$	0.301	${\left[\left[146,46,100/4;2\right]\right]}_{27^{2}}$	0.301
${\left[\left[146,138,98/4;92\right]\right]}_{27^{2}}$	0.315	${\left[\left[146,48,98/4;2\right]\right]}_{27^{2}}$	0.315
${\left[\left[146,144,96/4;96\right]\right]}_{27^{2}}$	0.329	${\left[\left[146,50,96/4;2\right]\right]}_{27^{2}}$	0.329
—	—	${\left[\left[146,52,94/4;2\right]\right]}_{27^{2}}$	0.343
—	—	${\left[\left[146,54,92/4;2\right]\right]}_{27^{2}}$	0.356
…	…	…	…
—	—	${\left[\left[146,138,12/4;2\right]\right]}_{27^{2}}$	0.932
—	—	${\left[\left[146,140,10/4;2\right]\right]}_{27^{2}}$	0.945
—	—	${\left[\left[146,142,8/4;2\right]\right]}_{27^{2}}$	0.959
—	—	${\left[\left[146,144,6/4;2\right]\right]}_{27^{2}}$	0.973

## Data Availability

Data are contained within the article.
